# Geogenic controls and anthropogenic inputs on the seasonal hydrogeochemical variability of the Paraopeba River basin, Brazil

**DOI:** 10.1007/s10653-026-03466-4

**Published:** 2026-10-12

**Authors:** Normara Yane Mar da Costa Andrade, Gabriel Soares de Almeida, Lúcio Cardoso de Medeiros Filho, Roberto Dall’Agnol, Prafulla Kumar Sahoo, Lucas Pereira Leão, Fernando Verassani Laureano, Vitor Brognaro Pimenta, Gabriel Negreiros Salomão

**Affiliations:** 1 Instituto Tecnológico Vale, Belém, PA Brazil; 2 Programa de Pós-Graduação em Geologia e Geoquímica (PPGG), Universidade Federal do Pará (UFPA), Instituto de Geociências, Belém, PA Brazil; 3 Department of Environmental Science and Technology, Central University of Punjab, Bathinda, Punjab India; 4 Departament of Geology, School of Mines, Federal University of Ouro Preto, Ouro Preto, MG Brazil; 5 Vale S/A, Diretoria de Reparação, Nova Lima, MG Brazil

**Keywords:** Hydrogeochemistry, Geochemical baseline, Surface water quality, Lithological control, Anthropogenic influence

## Abstract

This study investigates the combined effects of geology, land use and land cover, hydrological seasonality, and disturbance history on the hydrogeochemistry of surface waters in the Paraopeba River Basin (PRB), located in Minas Gerais State, southeastern Brazil, and forming part of the São Francisco River Basin, a region characterized by high lithological complexity and intense anthropogenic pressure. A total of 264 sites were sampled during the dry season (2022) and 262 during the rainy season (2023), allowing an integrated assessment of spatial and temporal variability. Hydrochemical facies are predominantly mixed bicarbonate, with water chemistry mainly controlled by silicate weathering. Localized deviations toward Na, Cl and SO_4_^2−^ enriched compositions are observed in urban and industrialized areas, reflecting anthropogenic contributions that are more pronounced during the dry season. In the confluence of the Paraopeba River with the Maranhão River it is observed a major hydrogeochemical control, resulting increased concentrations of Mn, Ba, SO_4_^2−^, Cl^−^, NO_3_^−^, and P downstream in the Paraopeba River, with persistent influence even under seasonal dilution conditions. The effects of the tailings related to the reservoir failure in Brumadinho, a municipality in central Minas Gerais, were evaluated; however, the analyzed dataset does not show a consistent downstream geochemical signature attributable to tailings remobilization. Current variability is predominantly controlled by diffuse and point-source inputs associated with land use. Geochemical reference values for Al, Fe, Mn, Ni, Ba, and Sr were established using the mMAD, TIF, and UTL methods within four integrated geospatial classes. The UTL method showed greater consistency in defining thresholds, approximating upper percentiles (P95–P98) or maximum values. Seasonal effects are more pronounced for Fe and Al, whereas Mn shows greater mobility in the dissolved phase. Overall, hydrogeochemical variability is primarily controlled by geology, modulated by hydrological seasonality, and intensified by anthropogenic pressure.

**Supplementary Information:**

The online version contains supplementary material available at https://doi.org/10.1007/s10653-026-03466-4.

## Introduction

Water quality in river basins results from the interaction between natural controls and anthropogenic pressure. Lithology and hydrological dynamics play a central role in hydrogeochemical processes in fluvial systems, whereas urbanization, agriculture, pasture, mining, and industrial development may significantly modify them (Alamri et al., 2021; Salomão et al., 2026; Tiwari et al., 2021). Changes in land use and land cover (LULC) affect surface runoff patterns, sediment transport, and diffuse solute inputs, directly influencing the chemical composition of surface waters (Calazans et al., 2018; Yang et al., 2020).

In tropical regions, strong hydrological seasonality intensifies chemical weathering, enhancing water–rock interactions and promoting spatial and seasonal variations in hydrogeochemical signatures. In this context, understanding the influence of landscape composition, spatial configuration, and precipitation on physicochemical parameters is essential to support effective conservation and management strategies at a watershed scale (Lei et al., 2021; Xiao & Ji, 2007). These variations modulate hydrogeochemical conditions and water quality patterns, with direct implications for food security, public health, biodiversity, and ecosystem services (Bila Júnior et al., 2024; Pant et al., 2021).

**Fig. 1:**
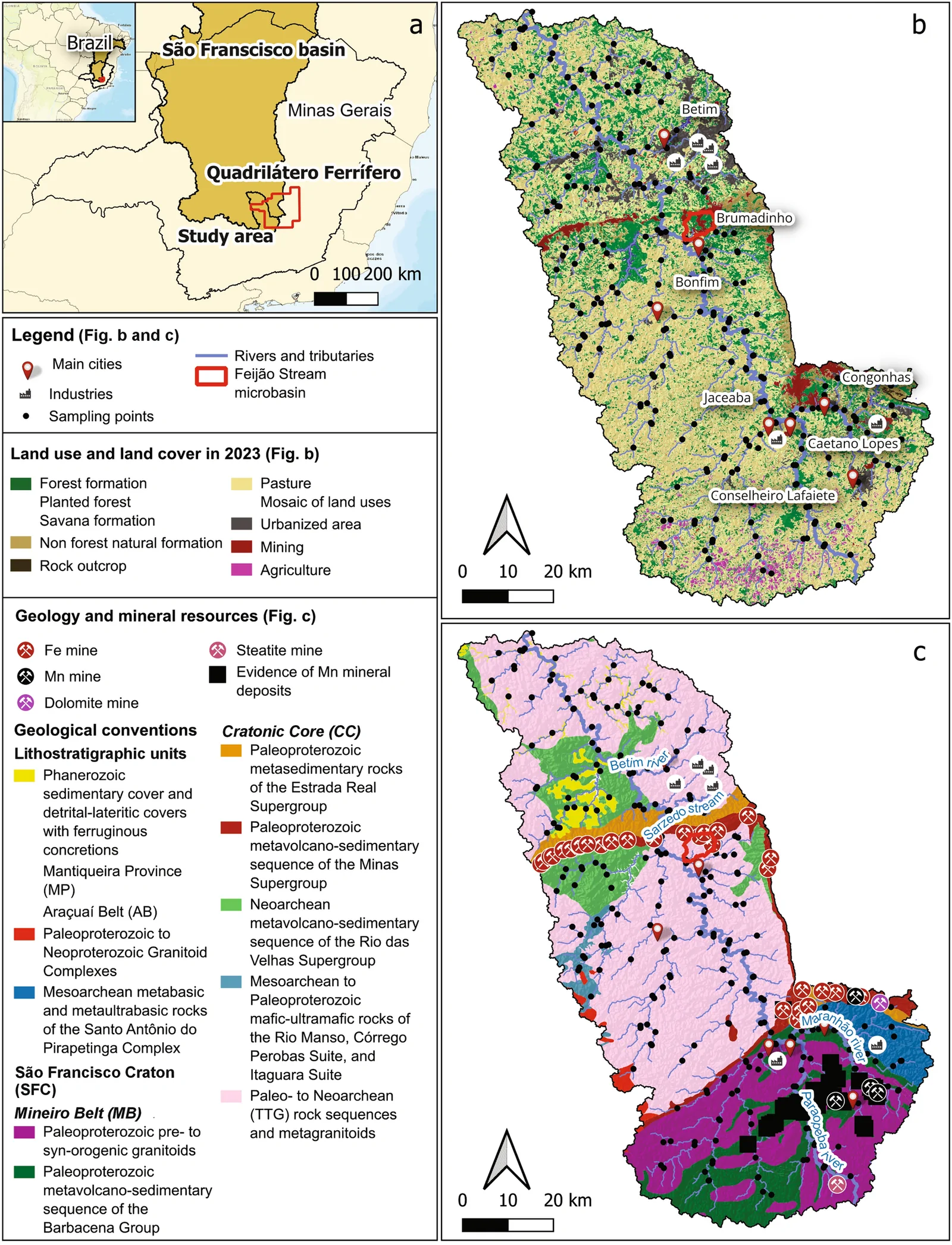
**a** Location of the study area and regional setting within the state of Minas Gerais, southeastern Brazil, highlighting the São Francisco River Basin. **b** Geological map of the study area (**c**) Land use and land cover in the study area, including the surface-water sampling network. The red square represents the microbasin associated with the sampling point located in the Feijão Stream, corresponding to the area where the iron ore tailings dam failure occurred in 2019

The distinction between geogenic and anthropogenic contributions remains a challenge in basins with high geological complexity and a long history of land occupation. Natural enrichment associated with specific lithologies may overlap with diffuse contamination, leading to misinterpretations when generic regulatory limits are applied without proper lithological conditioning (Costa et al., 2016; Salomão et al., 2021a, 2021b). In this context, the definition of geochemical reference values is essential to differentiate natural concentrations from those potentially influenced by anthropogenic activities. Considering the absence of fully preserved areas in the Paraopeba River Basin (PRB), located in Minas Gerais State, southeastern Brazil (Fig. 1a), and the fragmentation of the Atlantic Forest, and the fragmentation of the Atlantic Forest, the adoption of baselines that integrate natural contributions and diffuse anthropogenic inputs provides a more realistic representation of the current environmental state (Caritat et al., 2001; Matschullat et al., 2000; Reimann & Garrett, 2005). The estimation of such baselines requires the integration of lithology, geomorphology, soils, climate, biological activity, and land use, since broad regulatory standards often fail to capture local geochemical variability and may overestimate or underestimate environmental risks.

In this context, the PRB represents a key system for investigating the combined effects of geology, land use change, and hydrological seasonality on surface water chemistry. The PRB encompasses the Quadrilátero Ferrífero (QF), a globally significant metallogenetic province, where naturally elevated concentrations of Potentially Toxic Elements (PTEs) may occur due to the regional geogenic context (Almeida et al., 2025, 2026; Leão et al., 2019; Vicq et al., 2018, 2025). In addition, the basin has undergone intense and heterogeneous land use changes (MapBiomas, 2024), including urban expansion, industrial activities, agriculture, pasture, and mining, which exert distinct pressures on water quality.

Furthermore, on 2019 January 25th, the failure of the B1 tailings reservoirs at the Feijão Stream Mine Complex in Brumadinho, released approximately 12 million m^3^ of iron ore tailings into the Ferro-Carvão Stream, of which 1.6 Mm^3^ reached the Paraopeba River. The event caused extensive environmental impacts, including increased suspended sediment loads, deterioration of water quality, and changes in the hydrogeochemical conditions of the river system. It also intensified concerns regarding the transport, deposition, and potential remobilization of mine tailings in fluvial environment. Studies have reported initial increases in suspended sediments, turbidity, and metal concentrations, followed by progressive attenuation driven by dilution, sedimentation, and hydrological dynamics (Crioni et al., 2023; Lima et al., 2024; Pacheco et al., 2023). This behavior highlights the need for integrated basin-scale approaches capable of distinguishing transient disturbances from persistent hydrogeochemical patterns and separating natural variability from anthropogenic contributions.

The present study is part of the project “Geochemical Mapping and Context of the Quadrilátero Ferrífero” (GMBQF), conducted by the Instituto Tecnológico Vale (ITV), which provided an extensive regional geochemical dataset. Recent studies have addressed the geochemistry of surface waters (Almeida et al., 2025; Salomão et al., 2026) and stream sediments (Medeiros Filho et al., 2025); however, gaps remain in the understanding of multielement geochemical associations and in the definition of reference values based on surface waters in the PRB.

In this context, the objectives of this study are: (i) to evaluate the influence of geology and land use and land cover (LULC) on the geochemical characteristics and element distribution in surface waters of the PRB using GMBQF data to describe major ion patterns, hydrochemical facies, and potential contamination sources; (ii) to quantify spatial contrasts through geospatial classes integrating hydrography, geology, and land use; and (iii) to propose geochemical reference values for selected elements across the basin based on integrated geoprocessing and advanced statistical approaches.

## Study area

The Paraopeba River Basin covers approximately 13,643 km^2^ and the Paraopeba River extends for approximately 510 km before flowing into the Três Marias Reservoir, constituting one of the main tributaries of the São Francisco River (Santos et al., 2024). According to the Köppen–Geiger climate classification, the basin encompasses the Aw, Cwa, and Cwb climate types, with mean annual temperatures ranges from 19 to 23 °C and mean annual precipitation between 1200 and 1600 mm. Rainfall is strongly seasonal, with a rainy season extending from October to March and a dry season from April to September (Alvares et al., 2013; Bila Júnior et al., 2024). The study area is characterized by a highly heterogeneous LULC mosaic (Fig. 1b), where urban–industrial expansion, agriculture, and mining generate cumulative pressures that can alter surface water chemistry. Notably, the basin intersects one of the state’s major economic corridors and the broader Belo Horizonte metropolitan area), recognized as Brazil’s second-largest industrial hub. This area concentrates activities including metallurgy, food processing, and automobile manufacturing, along with mining, sand and clay extraction, cattle ranching, extensive livestock farming, eucalyptus monocultures, and other agricultural uses (Bila Júnior et al., 2024; Calazans et al., 2018; Lima et al., 2024).

The upper PRB, located in the southern portion of the study area, is predominantly characterized by mosaics of agricultural areas and pasturelands (Fig. 1b). Native vegetation and reforested areas are also present, although they are spatially fragmented. Iron mining activities occur mainly in the municipality of Congonhas, often in areas bordering urban zones (Fig. 1b).

In the middle course of the PRB, livestock farming represents one of the main land-use practices (Fig. 1b). However, near the Belo Horizonte Metropolitan Region, urbanization, industrialization, and infrastructure expansion intensify, including buildings, road networks, and recreational areas, while mining activities remain present within the metropolitan region (Fig. 1b).

The geological framework of the PRB is highly complex and consists mainly of Mesoarchean gneissic–granitoid complexes and migmatized tonalite–trondhjemite–granodiorite (TTG) cores, overlain by the Neoarchean sequence of the Rio das Velhas supergroup and the Paleoproterozoic Estrada Real and Minas supergroups (Fig. 1c). These supergroups comprise metavolcanosedimentary rocks, banded iron formations, phyllites, and quartzites and constitute the main regional metallogenetic framework, directly controlling the concentration of iron and manganese deposits extensively exploited along the structural axis of the Quadrilátero Ferrífero (Alkmim, 1998; Alkmim & Teixeira, 2016; Araújo et al., 2020; Rosière et al., 2008). The overlying Estrada Real Supergroup is composed of metasedimentary rocks, mainly phyllites and schists (metapelites) rich in quartz, sericite, chlorite, and biotite, commonly containing carbonaceous and/or graphitic material (Cavalcanti et al., 2023). The Mineiro Belt, associated with the Transamazonian Orogeny, includes granites, TTG gneisses, and metavolcanic rocks that display diverse geochemical signatures (Ávila et al., 2014).

## Materials and methods

### Sampling network design and analytical methods

Surface water samples were collected at 264 sites during the dry season in 2022 and 262 sites during the rainy season in 2023 (Fig. 1b, c). The difference in the number of sampling sites reflects the lack of access to two locations during the rainy season. Sampling and laboratory analyses were conducted by SGS Geosol LTDA (Vespasiano, MG). All procedures, from sample container preparation and field sampling to sample preservation, aliquot preparation, and laboratory analyses, followed the protocols described in the 23rd edition of the Standard Methods for the Examination of Water and Wastewater (APHA, 2017). In situ measurements of electrical conductivity, pH, dissolved oxygen, and temperature were obtained using a multiparameter probe (HANNA HI98194), and turbidity was measured with a turbidimeter. Laboratory analyses included the determination of anions (Cl^−^, F^−^, NO_2_^−^, NO_3_^−^, S^2^^−^, and SO_4_^2−^) by ion chromatography and total phosphorus by colorimetry. Total solids (TS), total dissolved solids (TDS), and suspended solids (SS) were quantified gravimetrically. Major and minor elements (Al, Mn, Fe) were analyzed by Inductively Coupled Plasma Optical Emission Spectroscopy (ICP-OES), while trace elements (Ag, As, Ba, Be, B, Cd, Co, Cr, Cu, Hg, Li, Ni, Pb, Sb, Se, U, V, and Zn) were determined by Inductively Coupled Plasma Mass Spectrometry (ICP-MS). Detailed descriptions of the sampling design, site selection criteria, quality assurance and quality control, and methodological framework are provided in Almeida et al. (2025).

### Geospatial analysis

Geospatial analysis involved the delineation of microbasins with an area of approximately 25 km^2^ for each sampling point, using digital elevation models (DEMs) derived from the ALOS Global Surface Model and hydrological modeling tools (Abdolmaleki et al., 2014; Spadoni, 2006; Spadoni et al., 2004). Geochemical distribution maps were generated based on these hydrographic units, representing the concentrations of individual elements in surface waters (Bonham-Carter et al., 1987; Carranza, 2011; Carranza & Hale, 1997; Salomão et al., 2018).

### Data processing, statistical analysis, and surface water characterization

The methodological procedures adopted in this study follow standard approaches widely applied in geochemical assessments (Calazans et al., 2018; Caritat et al., 2001; Caritat, P. de, 2017; Chai et al., 2020; Farias et al., 2021; Reimann & Garrett, 2005; Sahoo et al., 2020; Salomão et al., 2021a, 2021b; Salomão et al., 2025; Salomão et al., 2026). Initially, values below the limit of quantification (LQ) were replaced by half the LQ (LQ/2), whereas values exceeding the upper quantification limit were replaced by 1.5 times the LQ (LQ × 1.5) (Grunsky, 2010). The Shapiro Wilk test (Shapiro & Wilk, 1965) was used to assess the normality of data distribution. As most variables did not meet the assumption of normality, seasonal differences for each water-quality parameter were evaluated using the nonparametric Mann Whitney U test (Mann & Whitney, 1947), which compares the distributions of two independent groups without assuming a normal distribution.

**Fig. 2:**
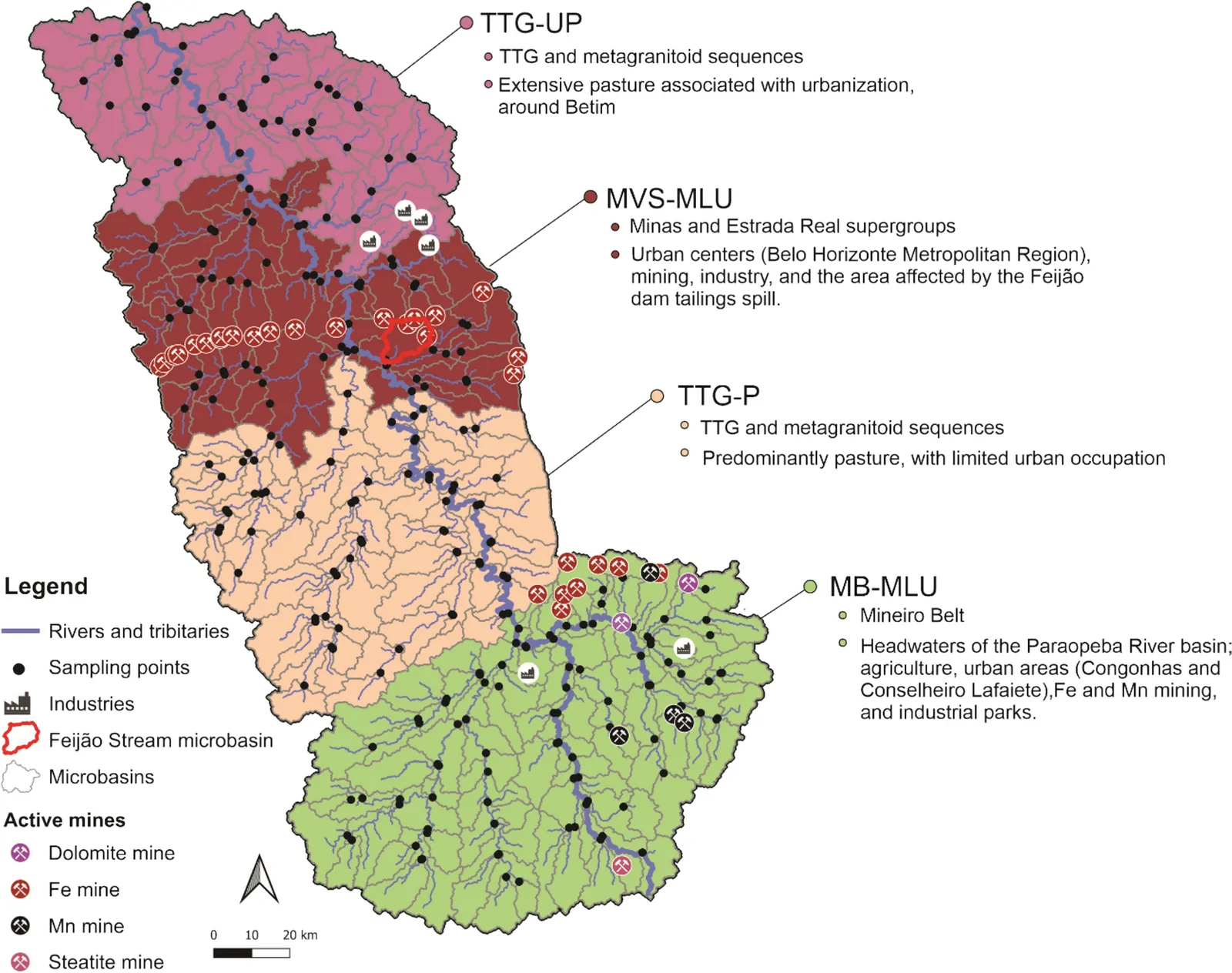
Schematic map of the grouping of microbasins according to the classes defined in the Paraopeba River basin. The legend illustrates the classification system adopted for the watershed. Each sampling point shown in Fig. 1 is assigned to one of four classes, which integrate geological attributes, land use and land cover, hydrological continuity, and territorial organization. The yellow square represents the microbasin associated with the sampling point located in the Feijão Stream, corresponding to the area where the iron ore tailings dam failure occurred in 2019

First, each site was classified based on its geochemical behavior associated with the dominant geological unit within the sampling site catchment, while LULC was employed as a complementary criterion to support environmental interpretation. This procedure is based on a study also conducted in the same region (Salomão et al., 2026). The resulting classes integrate geological attributes, land use and land cover, hydrological continuity, and territorial organization (Fig. 2), and are defined as follows:

(i) *Mineiro Belt–Multiple Land Use (MB-MLU), *which corresponds to the Mineiro Belt at the headwaters of the Paraopeba River basin and is characterized by diverse land uses, predominantly agricultural activities, associated with intense urbanization in municipalities such as Congonhas and Conselheiro Lafaiete, as well as the presence of mining areas, mainly of Fe and Mn, and industrial parks;
(ii) *TTG–Pasture Dominated (TTG-P),* which develops over TTG and metagranitoid sequences and is characterized by the predominance of pasture, with only limited occurrence of populated areas;
(iii) *MVS–Multiple Land Use (MVS-MLU),* which occurs around the mountain ranges associated with the Minas and Estrada Real supergroups and concentrates urban centers, including the Belo Horizonte Metropolitan Region, together with mining and industrial activities. This region also includes the area affected by the tailings related to the Feijão dam rupture; and.
(iv) *TTG–Urban and Pasture (TTG-UP),* which also occurs over TTG and metagranitoid sequences, but downstream of the mountain ranges associated with the MVS-MLU class and is characterized by the extensive distribution of pastures combined with urbanization, especially around the municipality of Betim.

Hydrochemical characterization employed the diagrams proposed by Gaillardet et al. (1999), Gibbs (1970), Piper (1944) and Schoeller (1980) to classify water types, identify dominant hydrogeochemical facies, and infer the main processes controlling water chemistry. These graphical approaches enabled the evaluation of the relative contributions of rock–water interactions, atmospheric inputs, and anthropogenic influences to the observed chemical composition. Molar ratios were ultimately expressed as median ± median absolute deviation (MAD). All statistical analyses and data processing were conducted in Python (Python Software Foundation, 2024), using the open-source package WQChartPy (Yang et al., 2022).

### Determination of geochemical baseline values

Baseline values were determined using widely recognized methods described in the literature (Ander et al., 2013; Nakić et al., 2007; Reimann et al., 2018; Reimann & Caritat, 2017; Sahoo et al., 2019; Salomão et al., 2018; USEPA, 2022a, 2022b). The applied approaches included Upper Tolerance Limits (UTL), Median + 2 × Median Absolute Deviation (mMAD), and the Tukey Inner Fence (TIF). These values were calculated using custom scripts developed in the open-source environment Python (Python Software Foundation, 2024), except for the UTL method, which was implemented using the software ProUCL (USEPA, 2022a, 2022b). Exploratory analyses supported the identification of distinct population types within the dataset, enabling the definition of reference values for specific areas based on their respective land-use characteristics. This approach allowed the proposal of realistic and geospatially representative reference values for the study area. Because the Paraopeba River Basin lacks fully preserved reference areas, no attempt was made to distinguish pristine from anthropogenically influenced sites. Instead, geochemical reference values were estimated for integrated geospatial classes representing the current environmental conditions of the basin, where both natural and diffuse anthropogenic contributions are present. Integrating lithology, land use and land cover, and hydrography into geospatial classes reduces the influence of geological heterogeneity and enables the reference values to reflect the dominant environmental conditions within each sector of the basin.

## Results and discussion

### Role of geology and land use in shaping surface water hydrogeochemistry

Understanding the hydrogeochemical characteristics of surface waters is essential for watershed protection and management. This study provides a hydrogeochemical characterization based on the four classes defined in the methodology, supported by general statistical tools applied to two hydrological cycles. The Piper diagram indicates that surface waters in the study area are predominantly of the mixed bicarbonate type during both dry and rainy periods (Piper, 1944). However, during the dry season, some samples exhibit a tendency toward enrichment in alkali metals (Na^+^ and K^+^) and chloride (Cl^−^). The Gaillardet diagram was used to assess lithological controls on water chemistry based on the normalized molar ratios HCO_3_^−^/Na^+^ and Mg^2^^+^/Na^+^ versus Ca^2^^+^/Na^+^ (Gaillardet et al., 1999). The results indicate that most samples are located between the silicate and carbonate domains, with a subset located in the evaporite field. This behavior is particularly evident in samples from the Betim River, Paraopeba River, and Maranhão River, which will be discussed in detail. The Gibbs diagram allows the identification of the main environmental processes controlling the chemical composition of surface waters, namely: (i) precipitation; (ii) rock weathering; and (iii) evapotranspiration and/or crystallization (Gibbs, 1970). Overall, the samples are situated between the weathering and precipitation domains. These findings are consistent with previous studies conducted across the entire QF region (Almeida et al., 2025), as well as with local-scale assessments (Paula et al., 2018; Salomão et al., 2026).

**Fig. 3:**
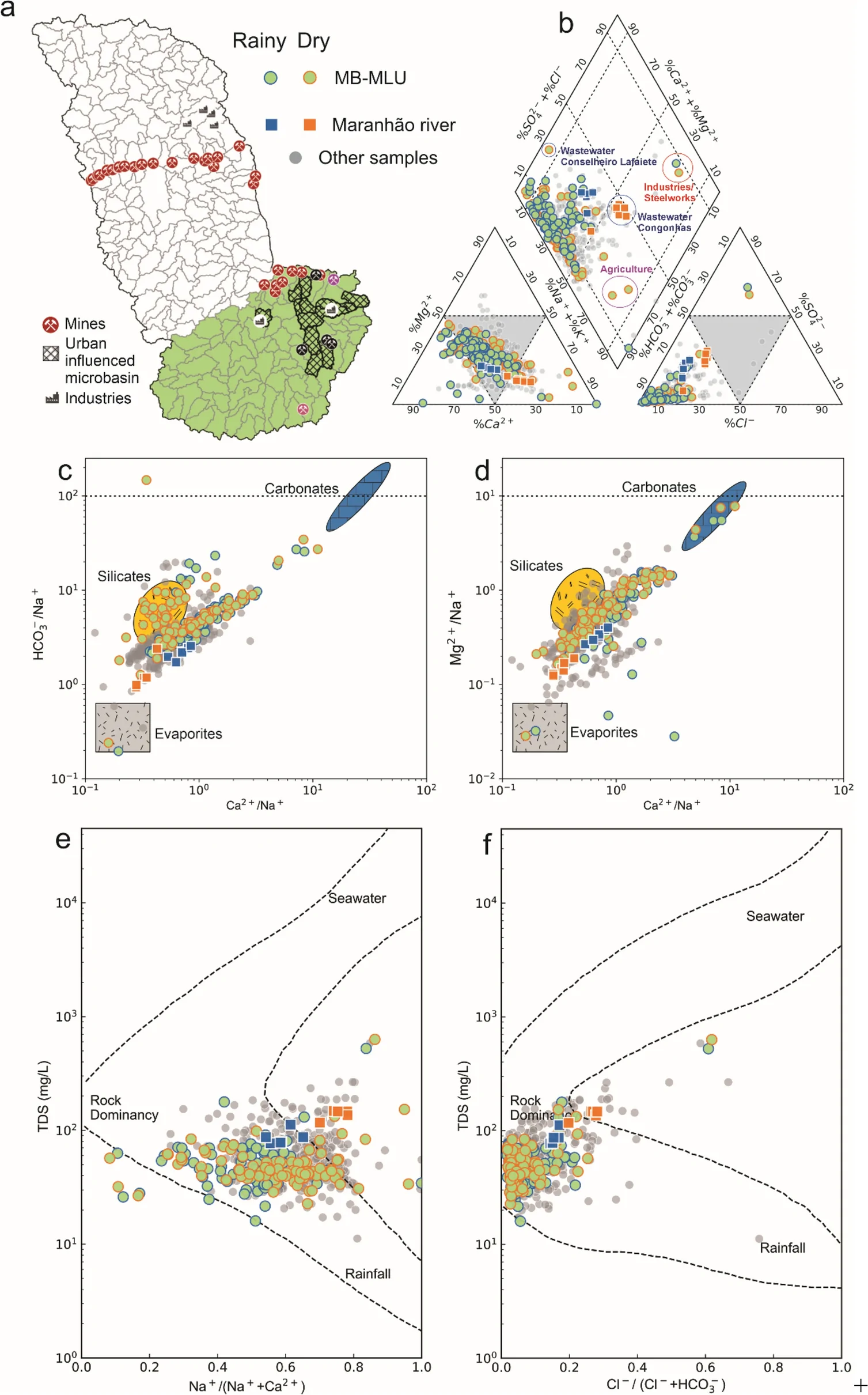
Hydrogeochemical characteristics of surface waters in the **a** MB-MLU class: **b** Piper diagram showing the main hydrochemical facies; **c**, **d** Gaillardet diagrams showing Na-normalized molar ratios in the dissolved phase; and (**e**, **f**) Gibbs diagrams illustrating molar ratios as a function of total dissolved solids (TDS)

In the MB–MLU class (Fig. 3a), the samples are predominantly of the calcium or magnesium bicarbonate type, as shown in Fig. 3b. However, a subset of samples is observed migrating toward the field of sulfated or sodium chloride waters, particularly during the dry season and at points associated with the Maranhão River. This trend suggests anthropogenic contributions, as these sampling sites are located within the MB–MLU class, which is characterized by intense urbanization, mining, and industrial parks, particularly in the cities of Congonhas and Conselheiro Lafaiete (Fig. 1b), In this setting, sewage discharge, urban runoff, and industrial activities represent sources of Na^+^, Cl^−^, and SO_4_^2−^ enrichment, as previously reported by Salomão et al. (2026).

The Gaillardet diagram (Fig. 3c, d) indicates a pronounced contribution from silicates and carbonate-rich rocks (Fig. 3c, d), as evidenced by the high Ca^2^^+^/Na^+^ ratios (1.06 ± 1.51 and 1.25 ± 1.22) and Mg^2^^+^/Na^+^ ratios (0.78 ± 1.17 and 0.80 ± 0.87). These waters also exhibit high HCO_3_^−^/Na^+^ ratios (9.24 ± 19.23 and 5.87 ± 5.04), reflecting water–rock interaction and dissolution processes involving CO₂ from the soil and atmosphere (Costa et al., 2016; Salomão et al., 2023, 2026). These patterns are consistent with the occurrence of talc-enriched carbonate and ultramafic rocks within the MB–MLU class (Fig. 1c; Fig. 3a), which may contain minerals such as calcite, dolomite, and magnesite (Neves et al., 2016; Oliveira et al., 2015; Parra et al., 2007; Roeser et al., 1980).

Conversely, samples with low Ca^2^^+^/Na^+^ and HCO_3_^−^/Na^+^ values, located near the evaporite field, reflect possible influences from non-lithological processes, such as the presence of industries and domestic effluents in the Congonhas region (Fig. 1b), as already indicated in the Piper diagram. The Gibbs diagram (Fig. 3e, f) shows that most samples remain close to the boundary between precipitation dominance and rock dominance fields, with a clear predominance of the latter (Fig. 3e, f). This is characterized by low TDS (62.5 ± 67.04 and 58.47 ± 55.16), moderate to high Na^+^/(Na^+^ + Ca^2^^+^) ratios (0.58 ± 0.18 and 0.50 ± 0.14), and predominantly low Cl^−^/(Cl^−^ + HCO_3_^−^) ratios (0.08 ± 0.09 and 0.08 ± 0.08). However, a subset of samples with higher TDS values and relative enrichment in Na^+^ and Cl^−^ is observed, shifting toward the evaporation/crystallization field. These samples are mainly associated with the dry season and locations with specific land uses, such as urban areas and steelmaking industries, indicating the influence of domestic and industrial effluents (Calazans et al., 2018; Costa et al., 2016; Paula et al., 2018).

**Fig. 4:**
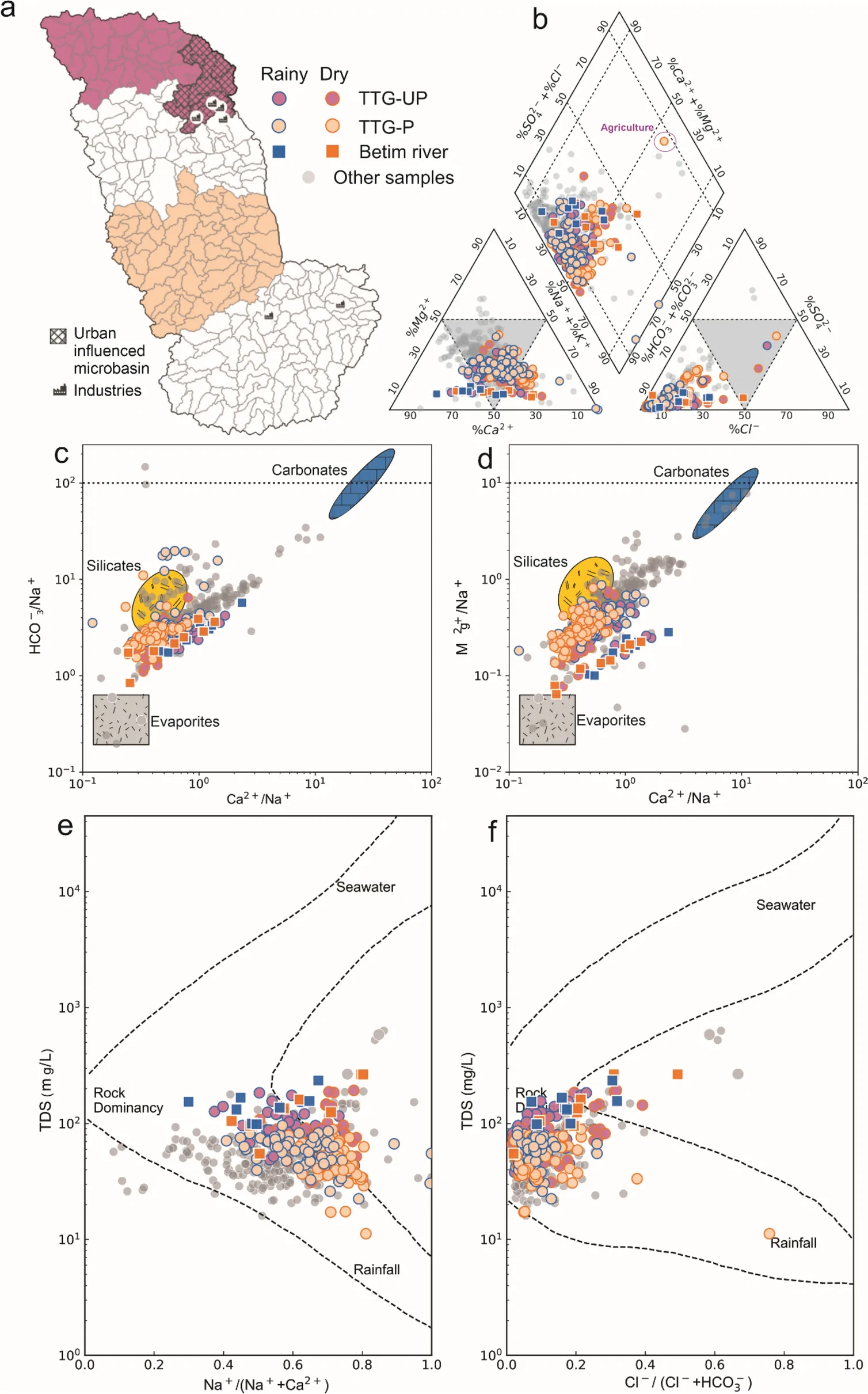
Hydrogeochemical characteristics of surface waters in the **a** TTG-UP and TTG-P classes: **b** Piper diagram showing the main hydrochemical facies; **c**, **d** Gaillardet diagrams showing Na-normalized molar ratios in the dissolved phase; and **e**, **f** Gibbs diagrams illustrating molar ratios as a function of total dissolved solids (TDS)

In the TTG–P and TTG–UP classes (Fig. 4a, b), the Piper diagram indicates a predominance of calcic or magnesian bicarbonate waters, with a tendency toward sodium bicarbonate compositions, reflecting the weathering of plagioclase-rich TTG and metagranitoid sequences that characterize the TTG–P and TTG–UP classes (Dorr, 1969; Rosière et al., 2008), with Na^+^ as the dominant cation and a significant contribution from Ca^2^^+^ in pasture areas. This pattern is consistent with the Gaillardet diagram (Fig. 4c, d), in which Ca^2^^+^/Na^+^ ratios vary between 0.40 ± 0.11 (TTG–P Dry) and 0.81 ± 0.37 (TTG–UP Rainy), while Mg^2^^+^/Na^+^ ratios (0.24 ± 0.14 for TTG–UP Dry and 0.39 ± 0.15 for TTG–P Rainy) remain lower, indicating a predominant contribution from plagioclase and a secondary influence of mafic minerals, mainly amphibole and biotite (Dorr, 1969). It is also observed that samples from the Betim River, along with other samples from the TTG–UP class, exhibit marked enrichment in Na^+^. In the Gibbs diagram (Fig. 4e,f), TTG–UP, as well as the Betim River, presents higher TDS concentrations compared to TTG–P. This pattern is consistent with the intense urbanization and industrialization of Betim (Fig. 1b), which has consolidated itself as one of the main petrochemical and automotive centers of the Metropolitan Region of Belo Horizonte (Delaunay et al., 2024; Howladar et al., 2017; Pant et al., 2021; Singh et al., 2008).

**Fig. 5:**
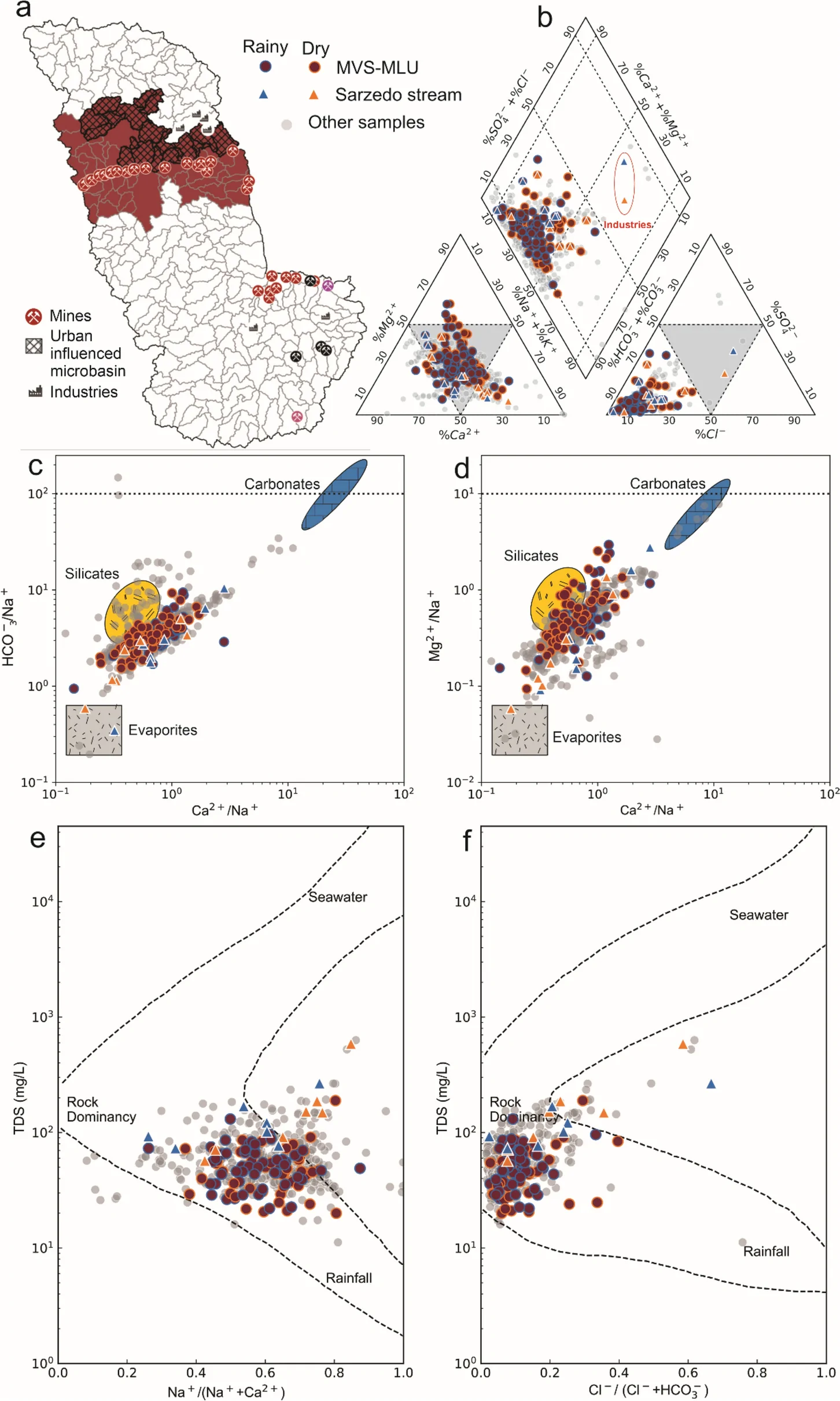
Hydrogeochemical characteristics of surface waters in the **a** MVS-MLU class: **b** Piper diagram showing the main hydrochemical facies; **c**, **d** Gaillardet diagrams showing Na-normalized molar ratios in the dissolved phase; and **e**, **f** Gibbs diagrams illustrating molar ratios as a function of total dissolved solids (TDS)

The MVS–MLU class reflects the lithological complexity of metavolcano-sedimentary units and the more diversified land use and occupation of the Paraopeba basin (Fig. 5a). In the Piper diagram (Fig. 5b), a predominance of calcic to mixed bicarbonate facies is observed. This variability is consistent with the mineralogical composition of the units of the Minas Supergroup and the Rio das Velhas Supergroup (Fig. 1c), which include dolomites and marbles (sources of Ca^2^^+^ and Mg^2^^+^), schists and phyllites with mica and chlorite (sources of Mg^2^^+^), as well as quartzites and itabirites (Endo et al., 2019; Oliveira et al., 2023; Raposo, 1991). In addition to lithological control, the MVS–MLU class encompasses areas of intense urbanization, industrial and mining activities in the Belo Horizonte Metropolitan Region (Fig. 1b), associated with elevated loads of organic matter (Bila Júnior et al., 2024; Paula et al., 2018; Silva et al., 2024; Sotiri et al., 2022). This effect is evidenced by the presence of samples showing a slight shift toward compositions richer in Na^+^, Cl^−^, and SO_4_^2−^ in the Piper diagram, as well as by locally higher TDS values.

The Gaillardet diagram (Fig. 5c, d) reflects this combination of controls, with Ca^2^^+^/Na^+^ ratios ranging from 0.68 to 0.85 and high Mg^2^^+^/Na^+^ ratios (0.62–0.68), indicating a simultaneous contribution from silicates, including some mafic minerals, and carbonates. The presence of carbonate lithologies (marbles and dolomite) within the MVS–MLU class (Fig. 5a) contributes to the elevation of Ca^2^^+^/Na^+^ and HCO_3_^−^/Na^+^ ratios, while ferromagnesian silicates, such as chlorite and biotite, explain the relatively high Mg^2^^+^/Na^+^ values compared to the TTG classes (Alkmim & Marshak, 1998; Alkmim & Teixeira, 2016; Araújo et al., 2020; Rosière et al., 2008).

The Gibbs diagram (Fig. 5e, f) corroborates the predominance of water–rock interaction control, with samples concentrated in the rock-dominant field and Na^+^/(Na^+^ + Ca^2^^+^) ratios ranging from 0.56 to 0.62, although with greater dispersion compared to the TTG classes. The TDS distribution, characterized by predominantly low to moderate values but with greater variability, indicates that the MVS–MLU class presents a complex hydrogeochemical signature resulting from the interaction between heterogeneous lithology and anthropogenic pressures.

### Hydrogeochemical evolution of the Paraopeba River driven by tributaries inputs

**Fig. 6:**
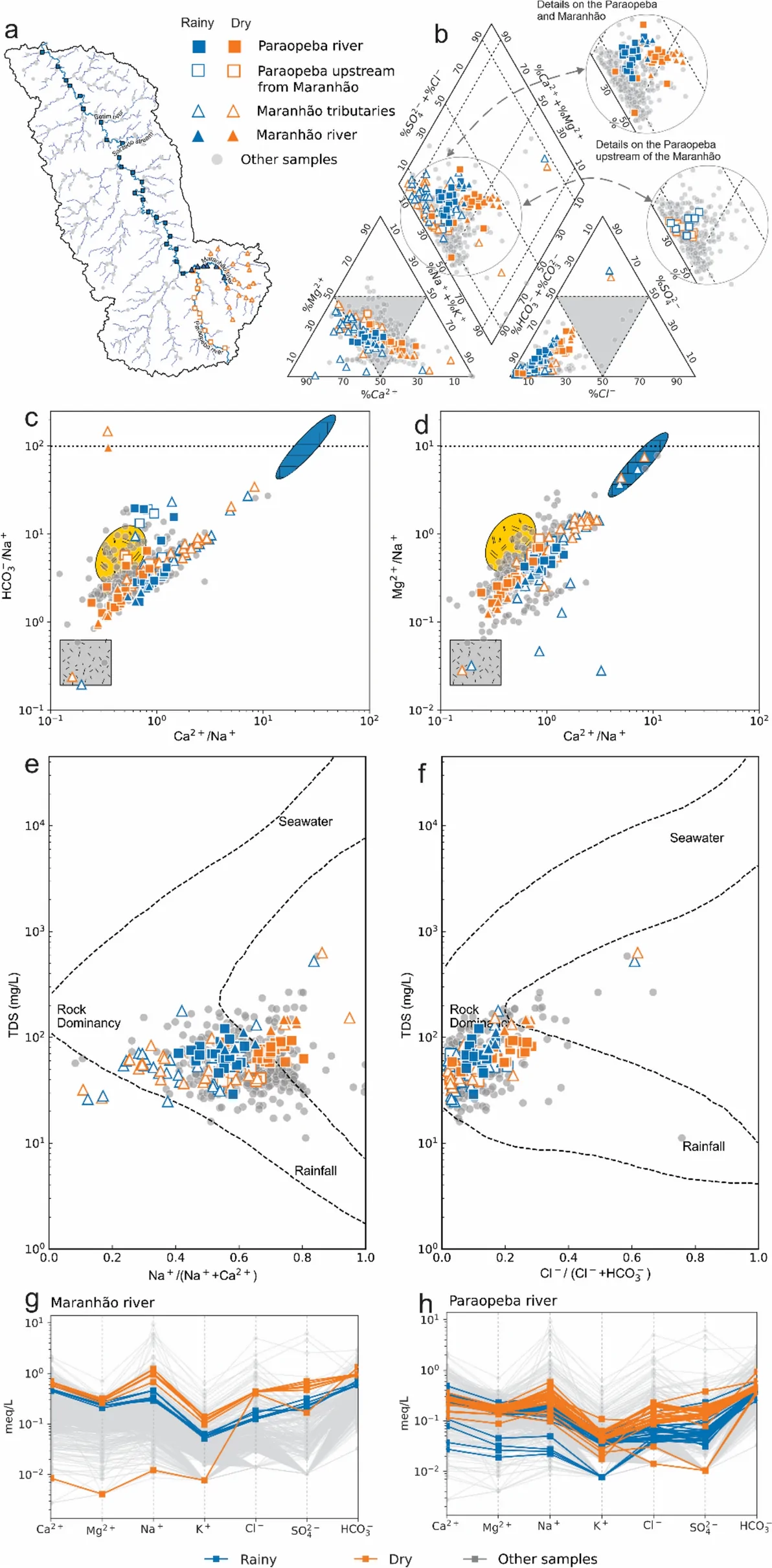
Hydrogeochemical characteristics of surface waters in the (**a**) Paraopeba River and Maranhão River: **b** Piper diagram showing the main hydrochemical facies; **c**, **d** Gaillardet diagrams showing Na-normalized molar ratios in the dissolved phase; **e**, **f** Gibbs diagrams illustrating molar ratios as a function of total dissolved solids (TDS); and **g**, **h** Schoeller diagrams comparing the dry and rainy periods in the Maranhão and Paraopeba rivers

In the basin drainage system, the Paraopeba River and the Maranhão River exhibit important hydrogeochemical behaviors. In the Piper diagram (Fig. 6b), both rivers show a predominance of calcium bicarbonate to mixed facies. However, the Maranhão River and a large portion of the samples from the Paraopeba River downstream of the confluence with the Maranhão River exhibit greater dispersion and a tendency to shift toward compositions enriched in Na^+^, Cl^−^, and SO_4_^2−^, reflecting the additional influence of anthropogenic sources, such as urbanization and mining within their drainage areas (Machado et al., 2024; Salomão et al., 2026). A similar trend is observed in samples from the dry period of the Paraopeba River downstream of the confluence with the Maranhão River, although to a lesser extent.

The Gaillardet diagrams (Fig. 6c, d) reinforce this differentiation, showing that samples from the Maranhão and Paraopeba rivers are predominantly concentrated near the silicate field. However, a systematic shift of samples toward the evaporite field is observed during the dry season. This shift is characterized by a reduction in the Ca^2^^+^/Na^+^, Mg^2^^+^/Na⁺, and HCO_3_^−^/Na^+^ ratios, reflecting relative enrichment in Na^+^ + K^+^ and Cl^−^, accompanied by a proportional decrease in Ca^2^^+^ and Mg^2^^+^. This shift is likely driven by increased contributions of domestic effluents in urbanized regions, notably the city of Congonhas (Calazans et al., 2018; Costa et al., 2016; Salomão et al., 2026). In contrast, tributaries of the Maranhão River show a shift toward the carbonate field, due to lithological contributions, including carbonate units discussed in the MB–MLU class (Oliveira et al., 2015; Roeser et al., 1980; Salomão et al., 2026).

In the Gibbs diagrams (Fig. 6e, f), both rivers remain predominantly within the rock-dominant field, with low to moderate TDS values. However, the Maranhão River exhibits greater variability and higher Na^+^/(Na^4^ + Ca^2^^+^) and Cl^−^/(Cl^−^ + HCO_3_^−^) ratios, especially during the dry season, indicating a stronger contribution of Na^+^ and Cl^−^.

The Schoeller diagrams (Fig. 6g, h) highlight these differences in greater detail, showing that the Maranhão River exhibits a wider amplitude and enrichment in Na^+^, K^+^, Cl^−^, and SO_4_^2^^−^, particularly during the dry season. This behavior reinforces the influence of anthropogenic sources, including urban runoff, domestic sewage, Fe and Mn mining, and industrial activities in the Congonhas and Conselheiro Lafaiete region, contributing to the greater hydrogeochemical complexity of the Maranhão River (Salomão et al., 2026).

Considering that the Paraopeba River is analyzed downstream of its confluence with the Maranhão River, its geochemical signature exhibits an intermediate character, reflecting the mixing between regional lithological control and the enrichment introduced by the Maranhão River. This pattern indicates that the Maranhão River acts as a vector of hydrogeochemical modification within the system, transferring its chemical signature to the Paraopeba River, where partial dilution occurs without complete attenuation of the geochemical signal.

**Fig. 7:**
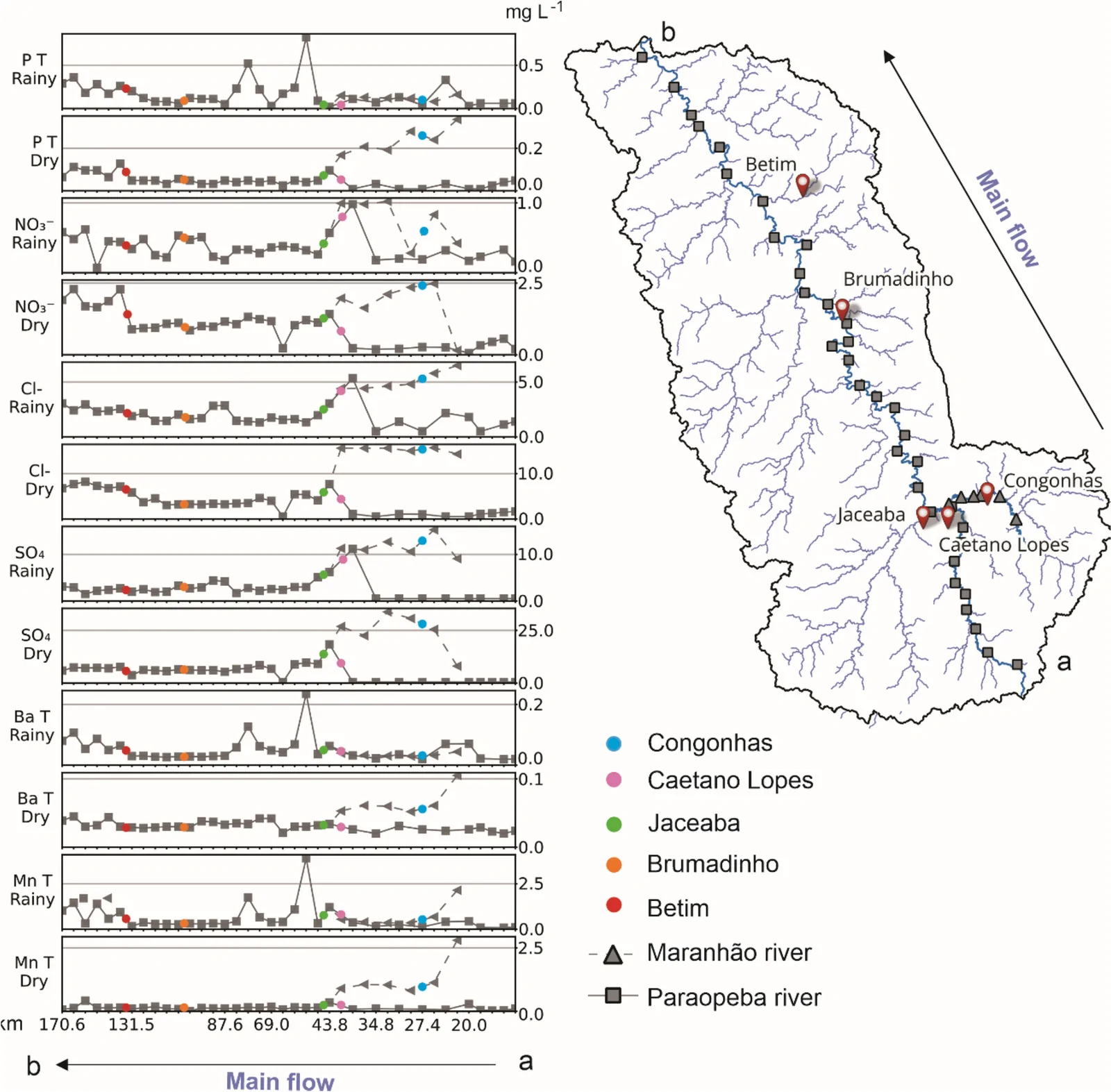
Longitudinal variation of Mn T, Ba T, SO_4_^2−^, Cl^−^, NO_3_^−^, and P T concentrations along the Paraopeba River (≈170 km), showing downstream changes influenced by the Maranhão River and by anthropogenic inputs associated with urban centers (Congonhas, Caetano Lopes, Jaceaba, Brumadinho, and Betim), under dry and rainy conditions

In this context, the longitudinal concentration profile (Fig. 7) reveals a well-defined geochemical change downstream of the confluence with the Maranhão River, marked by an increase in the concentrations of Mn, Ba, SO_4_^2−^, Cl^−^, NO_3_^−^, and P, as well as other parameters to a lesser extent. During the rainy season, a partial attenuation of these concentrations is observed, attributed to the hydrological dilution effect. However, even under higher discharge conditions, the concentrations recorded downstream remain systematically higher than those observed upstream in the Paraopeba River, indicating that the input from the Maranhão River exceeds the dilution effect and maintains its geochemical influence along the longitudinal profile (Mao et al., 2025; Mimura et al., 2016).

This change is directly associated with anthropogenic contributions to the Maranhão River basin (Salomão et al., 2026). In these sectors, the enrichment in Na^+^, Cl^−^, SO_4_^2^^−^ and nutrients (NO_3_^−^ and P) suggests the combined influence of domestic effluents and urban runoff, which significantly modify the chemical composition of the waters and shift the system towards signatures enriched in soluble ions (Duffus, 2002; Ngoye & Machiwa, 2004). Similarly, high concentrations of Mn are associated with the Conselheiro Lafaiete region, which harbors several deposits of this element (Salomão et al., 2026), increasing the contribution to the Paraopeba River downstream.

A similar behavior is observed in the Paraopeba River, downstream from its confluence with the Betim River, in the city of Betim (Fig. 7), where there is a further increase in the concentrations of Mn, Ba, SO_4_^2−^, Cl^−^, NO_3_^−^, and P, NO3^−^, indicating the influence of additional sources of predominantly urban and industrial origin in the region (Candeias et al., 2015; Huang et al., 2016). This behavior reinforces the role of the impacted tributaries as secondary controls of longitudinal variability, whose influence becomes more pronounced under low flow conditions.

Additionally, the absence of a marked geochemical signature in the Paraopeba River downstream of Brumadinho, directly associated with the 2019 dam failure, should be interpreted in light of the temporal evolution of impacts described in the literature (Lima et al., 2024; Pacheco et al., 2023; Silva et al., 2024; Teramoto et al., 2021). Studies conducted immediately after the event documented severe changes in water quality, including substantial increases in turbidity, suspended solids, and metal concentrations, such as Fe, Mn, and Al, particularly in the first kilometers downstream of the failure point (Pacheco et al., 2023; Silva et al., 2024). However, long-term monitoring studies indicate that these impacts were progressively attenuated in the water column, with reductions in turbidity, suspended solids, and dissolved metal concentrations, reflecting deposition, dilution, and fluvial system reorganization processes (Lima et al., 2024). Seasonal variability also plays a central role in this behavior, as hydrological events, particularly during the rainy season, promote the resuspension of previously deposited sediments and temporarily influence water quality (Costa et al., 2016; Salomão et al., 2026).

In this context, more recent investigations have shown that the water quality of the Paraopeba River reflects a multi-causal scenario in which different sources and processes coexist and interact throughout the basin, including urban areas, mining, industrial activities, livestock farming, agriculture, and the regional geological influence (Bila Júnior et al., 2024; Calazans et al., 2018; Menezes et al., 2025). Recent reviews also indicate that inputs from urbanized, industrial, and agricultural areas can significantly contribute to contaminant loads and may mask the geochemical imprint of the tailings from the dam, making it difficult to distinguish their influence along the fluvial system (Bila Júnior et al., 2024; Menezes et al., 2025; Pacheco et al., 2025). The absence of a consistent increase in Mn, Fe, and Ba concentrations (Fig. 7), together with the lack of a downstream enrichment pattern from the Feijão Stream, suggests that, at the analytical scale adopted in this study, the geochemical signature associated with the tailings released by the dam failure could not be robustly distinguished. Instead, the current hydrogeochemical variability of the Paraopeba River appears to be predominantly controlled by diffuse and point-source inputs related to land use and land cover, particularly in tributaries under strong urban, industrial, and mining influence (Almeida et al., 2025; Paula et al., 2018; Salomão et al., 2026), whereas the direct contribution of the 2019 event does not emerge as a dominant or distinctive factor.

### Determination of geochemical baseline values by hydrogeochemical classes and its environmental applications

Defining baseline and reference hydrogeochemical parameters is a crucial step in supporting water resource management in mineralized areas with diverse uses. These values are essential for distinguishing naturally elevated concentrations from those influenced by anthropogenic pressures (Caritat et al., 2001; Chai et al., 2020; Medeiros Filho et al., 2025; Reimann & Garrett, 2005; Salomão et al., 2026). Geochemical reference values incorporate both natural contributions and diffuse anthropogenic inputs, thereby representing the overall hydrogeochemical condition of the basin (Albanese et al., 2007; Hinsby et al., 2008; Salminen & Gregorauskien, 2000). The exploratory analyses applied in this study ("Role of geology and land use in shaping surface water hydrogeochemistry", and "Hydrogeochemical evolution of the Paraopeba River driven by tributaries inputs" section) were essential for characterizing spatial and seasonal patterns, identifying key geochemical indicators, and supporting the definition of reliable and spatially representative reference values.

In this study, reference values for Al, Ba, Fe, Mn, Ni, and Sr were established using complementary statistical approaches (UTL, mMAD, and TIF; Table 1) and integrated into spatial distribution mapping. This strategy refined the hydrogeochemical characterization of surface waters and strengthened the environmental interpretation of the dataset. The geochemical baselines were derived from samples classified as TTG-UP, MVS-MLU, TTG-P, and MB-MLU, as defined in "Data processing, statistical analysis, and surface water characterization"section. Table 1 compares the proposed reference levels among the geospatial classes.

**Table 1:** Baseline concentrations for the TTG-UP (n = 46), MVS-MLU (n = 58), TTG-P (n = 68), and MB-MLU (n = 92) classes

Parameter	Season	CONAMA 357/05 Class 2	CONAMA 357/05 Class 3	TTG-UP			MVS-MLU			TTG-P			MB-MLU		
				UTL	mMAD	TIF	UTL	mMAD	TIF	UTL	mMAD	TIF	UTL	mMAD	TIF
Al T	DS	–	–	1.48	0.84	1.54	0.46	0.79	1.97	0.59	0.55	1.49	0.72	0.33	1.01
	RS			13.43	17.59	56.32	5.51	0.91	1.92	20.23	21.65	76.38	25.1	8.47	47.3
Ba D	DS	–	–	0.18	0.09	0.11	0.06	0.07	0.1	0.04	0.04	0.06	0.07	0.07	0.10
	RS			0.1	0.11	0.16	0.07	0.04	0.06	0.07	0.04	0.06	0.07	0.04	0.05
Ba T	DS	0.7	0.7	0.88	0.12	0.15	0.07	0.09	0.12	0.04	0.06	0.06	0.12	0.06	0.07
	RS			0.12	0.12	0.14	0.07	0.07	0.11	0.29	0.05	0.06	0.15	0.06	0.09
Fe D	DS	0.3	5	1.37	1.2	1.63	0.85	1.1	1.61	0.62	0.71	0.87	1.17	0.75	1.35
	RS			1.82	3.35	4.43	1.21	1.55	2.23	1.45	1.95	4.36	1.64	1.7	2.5
Fe T	DS	–	–	5.01	3.4	4.02	4.05	2.28	3.4	1.72	1.81	2.15	3.56	1.94	2.16
	RS			11.09	7.49	10.71	5.93	6.28	11.81	45.4	9.53	16.74	19.9	9.35	14.84
Mn D	DS	–	–	0.35	0.36	0.51	0.68	0.37	0.62	0.23	0.16	0.32	2.54	0.47	0.75
	RS			0.51	0.18	0.23	0.6	0.51	0.81	1.97	0.65	0.63	1.19	0.53	1.72
Mn T	DS	0.1	0.5	0.39	0.43	0.84	1.03	0.57	0.87	0.25	0.11	0.25	3.51	0.63	0.94
	RS			1.45	0.3	0.63	0.65	0.80	1.43	3.90	0.41	0.63	0.97	0.87	1.94
Ni D	DS	–	–	0.05	0.05	0.09	0.03	0.03	0.06	0.02	0.02	0.03	0.03	0.03	0.05
	RS			0.04	0.06	0.08	0.03	0.04	0.06	0.03	0.05	0.11	0.03	0.04	0.06
Ni T	DS	0.025	0.025	0.12	0.08	0.11	0.06	0.07	0.08	0.05	0.05	0.07	0.05	0.05	0.07
	RS			0.24	0.17	0.2	0.14	0.16	0.28	1.32	0.18	0.26	0.46	0.19	0.28
Sr D	DS	–	–	0.32	0.18	0.37	0.11	0.14	0.23	0.07	0.07	0.11	0.41	0.14	0.22
	RS			0.22	0.3	0.38	0.11	0.11	0.16	0.06	0.07	0.10	0.39	0.08	0.10
Sr T	DS	–	–	1.17	0.18	0.36	0.12	0.14	0.25	0.67	0.08	0.19	1.46	0.08	0.11
	RS			0.23	0.29	0.45	0.12	0.12	0.17	0.08	0.07	0.10	0.10	0.07	0.08

Concentrations are expressed in mg L^−1^. The UTL methods implemented in ProUCL are described in online Appendix B

Threshold values were calculated for the dry and rainy seasons using the UTL, median + 2 × median absolute deviation (mMAD), and Tukey inner fence (TIF) methods

Each method used to derive baseline values presents specific strengths and limitations within its statistical framework. The mMAD approach is widely recognized as a robust strategy for outlier detection, as it shows low sensitivity to extreme values and high stability under skewed distributions, being widely applied in geochemical mapping (Matschullat et al., 2000; Mimba et al., 2018; Reimann et al., 2018; Sahoo et al., 2025).

The TIF method is based exclusively on the distributional structure of the dataset and allows the estimation of background values even when the dataset does not contain identifiable outliers (Reimann et al., 2018). In this approach, the estimated upper limit may exceed the maximum observed value, reflecting the theoretical behavior of the fitted distribution (Ahn et al., 2022; Sahoo et al., 2020).

The UTL method follows a probabilistic framework and depends directly on statistical modeling, assuming normal, lognormal, gamma, or non-parametric distributions (USEPA, 2022a). Consequently, its robustness and representativeness depend on how well the selected statistical model fits the empirical behavior of the dataset.

Among the evaluated methods (mMAD, TIF, and UTL), the UTL approach showed greater consistency with the statistical structure of the data and with the dominant geochemical processes in the Paraopeba River basin. Its reference values approximate the upper percentiles (≈P95–P98), do not exceed the observed maximum, and represent the recurrent upper concentration range in the basin. In contrast, the mMAD and TIF methods produced estimates that, in some cases, exceeded the maximum observed concentrations in the dataset (online Appendix A), which may lead to overestimation of reference levels.

Salomão et al. (2026) reported similar behavior in a study based on high-frequency environmental monitoring, in which statistical limits derived using mMAD and TIF also exceeded empirically observed values. Clarifying this pattern requires further investigation, as it may reflect the presence of undetected outliers, the effects of data transformations, or inherent statistical limitations of the dataset, including sample size, pronounced skewness, or inadequate fitting of the selected distribution model (Almeida et al., 2025; Salomão et al., 2025).

**Fig. 8:**
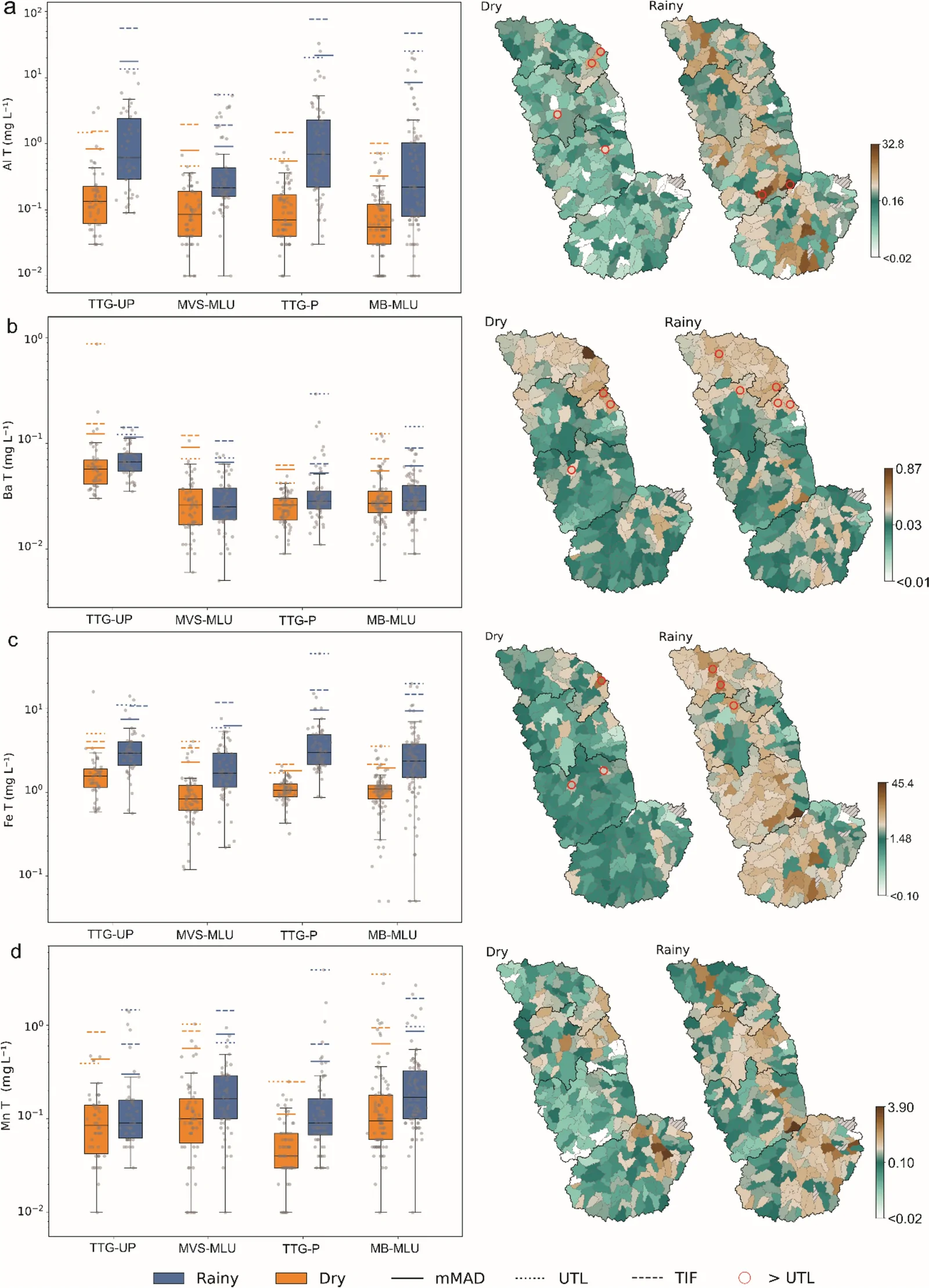
Box plots show all measurements from sampling points grouped according to their respective classes (Fig. 2). The different line types represent the baseline values derived from the mMAD, TIF, and UTL methods. The figure also presents spatial distribution maps of total Al, Ba, Fe, and Mn for the dry and rainy seasons

The UTL results indicate that, for the total fraction of Al and Fe, concentrations consistently increase during the rainy season across all classes. These elements characterize the rainy season, particularly in regions dominated by pasture, agriculture, and/or mixed land use (TTG-UP, TTG-P, and MB-MLU – Fig. 8a,c).The observed pattern indicates intensified mobilization of particulate material due to the absence of vegetation cover, reflected in increased suspended solids and turbidity, which promote the transport of Fe and Al oxides and clay minerals along the fluvial system (Costa et al., 2016; Pacheco et al., 2023; Salomão et al., 2023). In TTG-UP, Fe T increases from 5.1 mg L^−1^ in the dry season (DS) to 11.09 mg L^−1^ in the rainy season (RS), while Al T increases from 1.48 mg L^−1^ to 13.43 mg L^−1^. These reference values were used to identify high-concentration samples in boxplots and to support the interpretation of their spatial distribution (Fig. 8). Maps and boxplots for Ba D, Fe D, Mn D, Ni D, Ni T, Sr D, and Sr T are provided in online Appendix C.

Regarding Mn, the MB-MLU class, located in the headwaters of the basin, presents the highest total and dissolved Mn values during the dry season, with UTL values of 2.57 mg L^−1^ for Mn D and 3.51 mg L^−1^ for Mn T. These results are consistent with the geological and anthropogenic characteristics of the area, as several indications of manganese mineralization occur near Conselheiro Lafaiete. In addition, mining activity in Congonhas and intense urbanization in the region may act as additional sources or remobilization mechanisms, contributing to increased concentrations in this class (Salomão et al., 2026).

The distribution of Ba in (Fig. 8b) indicates that, although its origin is largely associated with the weathering of granitoid-gneissic and TTG units of the Quadrilátero Ferrífero basement, its concentration in surface waters are also modulated by land use and seasonality. Ba is a large-ion lithophile element with a tendency to concentrate in K-bearing minerals, such as alkali feldspar and biotite, particularly in felsic rocks (Deer et al., 2013). In the QF context, areas classified as TTG include not only tonalites and tonalitic orthogneisses but also granodiorites, monzogranites, leucogranites, migmatites, and granitic gneisses, reflecting a heterogeneous granitoid-gneissic domain with variable potential for Ba contribution.

However, as the TTG-UP and TTG-P classes share a similar lithological basis, the differences observed in the boxplots cannot be attributed exclusively to geological control. The higher values and greater dispersion in TTG-UP, especially during the rainy season, suggest enhanced mobilization of Ba associated with surface runoff in areas affected by urbanization, mining, and/or industrial activities. In the maps, the concentration of values above the UTL in microbasins under greater anthropogenic pressure, more evident during the rainy season, reinforces the role of seasonality in the remobilization of particulate material and the transport of trace elements (Viers et al., 2009). Thus, the whole data (Fig. 8b) indicate a mixed control, in which Ba has a geogenic origin associated with the granitoid-gneissic/TTG basement, while its spatial and seasonal variability is amplified by the interaction between land use and hydrological dynamics.

## Conclusions

This study presents an integrated hydrogeochemical assessment of the Paraopeba River Basin, highlighting the combined influence of geology, land use and land cover, hydrological seasonality, and disturbance history on the composition of surface waters.

- Lithological control is identified as the primary factor governing hydrogeochemical signatures across the basin, with silicate weathering as the dominant process and secondary contributions from carbonates. Surface waters are predominantly of the mixed bicarbonate type; however, localized enrichment in Na^+^, Cl^−^, and SO_4_^2−^ indicates anthropogenic inputs associated with urbanization, industrial activities, and mining, particularly during low-flow conditions.
- The Maranhão River acts as a major hydrogeochemical control, promoting significant changes in the composition of the Paraopeba River downstream, with increased concentrations of major ions, nutrients, and trace elements. This influence persists even during the rainy season, indicating that dilution processes are insufficient to fully attenuate the geochemical signal introduced by this tributary. Contributions from other impacted tributaries, such as the Betim River, further reinforce the role of land use in controlling longitudinal variability.
- Regarding the 2019 B1 tailings reservoirs failure at the Feijão Stream mine in Brumadinho, the results indicate that, at the spatial and temporal scale of this study, there is no evidence of a persistent or dominant hydrogeochemical signature in the water column attributable to the continuous remobilization of those tailings. Current variability is predominantly controlled by diffuse and point-source inputs associated with land use.
- Seasonality exerts a strong influence on element transport and partitioning. Increased concentrations of total Fe and Al during the rainy season reflect enhanced mobilization of suspended sediments, whereas Mn shows greater mobility in the dissolved phase, suggesting control by redox processes.
- The establishment of geochemical reference values based on integrated geospatial classes proved essential for distinguishing natural variability from anthropogenic influences. Among the evaluated methods, the UTL demonstrated greater robustness in defining thresholds, approximating upper percentiles (P95–P98) and avoiding the overestimation observed in the mMAD and TIF methods.

Overall, hydrogeochemical variability in the Paraopeba River Basin is primarily controlled by geological inheritance, modulated by hydrological seasonality, and locally intensified by land use. These results demonstrate that, in complex and historically impacted basins, water quality reflects the interaction of multiple factors, and that the adoption of reference values conditioned by geology and land use constitutes a more realistic approach for environmental monitoring and management.

## Supplementary Information

Below is the link to the electronic supplementary material.

Supplementary file1 (DOCX 2039 KB)

## Data Availability

Data will be made available upon request.
