# Supplementary material for: Geogenic controls and anthropogenic inputs on the seasonal hydrogeochemical variability of the Paraopeba River basin, Brazil
Source: Environ Geochem Health. 2026 Oct 12;48(16):634. doi: 10.1007/s10653-026-03466-4 (PMC13658292; doi:10.1007/s10653-026-03466-4)
Supplement: Supplementary file 1 [file 10653_2026_3466_MOESM1_ESM.docx]

**Appendices**

**Manuscript Title:** Geogenic controls and anthropogenic inputs on the seasonal hydrogeochemical variability of the Paraopeba River basin, Brazil

**Authors:** Normara Yane Mar da Costa Andrade, Gabriel Soares de Almeida, Lúcio Cardoso de Medeiros Filho, Roberto Dall’Agnol, Prafulla Kumar Sahoo, Lucas Pereira Leão, Fernando Verassani Laureano, Vitor Brognaro Pimenta, and Gabriel Negreiros Salomão^*^

***Corresponding author email:** gabriel.salomao@itv.org

**List of Appendices**

[Appendix A – Descriptive statistics of water quality parameters grouped by geospatial classes	2](#_Toc228879634)

[Appendix B – UTL methods	13](#_Toc228879635)

[Appendix C – Spatial distribution maps	17](#_Toc228879636)

# Appendix A – Descriptive statistics of water quality parameters grouped by geospatial classes

The p-value of the Mann-Whitney Test (pMW) was used to assess the statistical difference between the rainy (RS) and dry (DS) seasons. p-value of the Shapiro-Wilk (pSW) for normality test; number of samples (n); percentage of samples below the limit of quantification (%<LQ); arithmetic mean (µ); standard deviation (s); coefficient of variation (%CV); median absolute deviation (MAD); minimum (Min); percentile values (P25, P50, P75, P90, P95, and P98); maximum (Max), skewness, and kurtosis. For metals, ‘T’ denotes to total concentration, and ‘D’ to dissolved concentration.

## TTG-UP

| **Parameter** | **pMW** | **Dataset** | **pSW** | **n** | **% <LD** | **µ** | **s** | **% CV** | **MAD** | **Min** | **P25** | **P50** | **P75** | **P90** | **P95** | **P98** | **Max** | **skewness** | **kurtosis** |
| --- | --- | --- | --- | --- | --- | --- | --- | --- | --- | --- | --- | --- | --- | --- | --- | --- | --- | --- | --- |
| Alk HCO₃ | 0.09 | DS | <0.05 | 46 | 0 | 51.45 | 38.15 | 74.16 | 11.9 | 15.3 | 28.3 | 39.2 | 63.1 | 88.15 | 138 | 177.3 | 180 | 2.10 | 7.37 |
| Alk HCO₃ | 0.09 | RS | <0.05 | 46 | 0 | 38.84 | 21.42 | 55.14 | 12.55 | 13.9 | 22.575 | 34.55 | 48.1 | 67.35 | 80.5 | 99.12 | 102 | 1.29 | 4.44 |
| Alk T | 0.09 | DS | <0.05 | 46 | 0 | 51.45 | 38.15 | 74.16 | 11.9 | 15.3 | 28.3 | 39.2 | 63.1 | 88.15 | 138 | 177.3 | 180 | 2.10 | 7.37 |
| Alk T | 0.09 | RS | <0.05 | 46 | 0 | 38.84 | 21.42 | 55.14 | 12.55 | 13.9 | 22.575 | 34.55 | 48.1 | 67.35 | 80.5 | 99.12 | 102 | 1.29 | 4.44 |
| Al D | <0.05 | DS | <0.05 | 46 | 13.04 | 0.06 | 0.07 | 106.94 | 0.025 | <0.02 | 0.03 | 0.045 | 0.07 | 0.105 | 0.2025 | 0.322 | 0.34 | 2.88 | 11.92 |
| Al D | <0.05 | RS | <0.05 | 46 | 17.39 | 0.18 | 0.18 | 103.51 | 0.09 | <0.02 | 0.04 | 0.115 | 0.245 | 0.435 | 0.515 | 0.722 | 0.74 | 1.47 | 4.84 |
| Al T | <0.05 | DS | <0.05 | 46 | 0 | 0.33 | 0.66 | 202.68 | 0.075 | 0.03 | 0.0625 | 0.135 | 0.225 | 0.535 | 0.89 | 3.023 | 3.5 | 4.08 | 20.02 |
| Al T | <0.05 | RS | <0.05 | 46 | 0 | 1.87 | 2.77 | 148.27 | 0.48 | 0.09 | 0.29 | 0.62 | 2.405 | 4.405 | 7.575 | 11.75 | 12.2 | 2.52 | 9.61 |
| Ba D | 0.59 | DS | <0.05 | 46 | 0 | 0.05 | 0.03 | 53.73 | 0.0105 | 0.015 | 0.038 | 0.047 | 0.059 | 0.085 | 0.108 | 0.137 | 0.182 | 2.56 | 11.44 |
| Ba D | 0.59 | RS | <0.05 | 46 | 0 | 0.05 | 0.02 | 39.43 | 0.014 | 0.022 | 0.0365 | 0.053 | 0.065 | 0.075 | 0.082 | 0.0939 | 0.129 | 1.02 | 5.53 |
| Ba T | <0.05 | DS | <0.05 | 46 | 0 | 0.08 | 0.12 | 155.16 | 0.0135 | 0.03 | 0.041 | 0.057 | 0.070 | 0.101 | 0.135 | 0.267 | 0.875 | 6.13 | 42.75 |
| Ba T | <0.05 | RS | <0.05 | 46 | 0 | 0.07 | 0.02 | 34.16 | 0.0135 | 0.035 | 0.055 | 0.0665 | 0.08 | 0.104 | 0.111 | 0.1347 | 0.141 | 1.04 | 4.04 |
| Ca D | 0.64 | DS | <0.05 | 46 | 0 | 9.53 | 7.99 | 83.84 | 2.925 | 2.49 | 3.8925 | 6.52 | 14.5 | 19.45 | 21.45 | 25.91 | 43.1 | 2.04 | 8.61 |
| Ca D | 0.64 | RS | <0.05 | 46 | 0 | 9.68 | 6.82 | 70.47 | 3.335 | 2.46 | 4.1275 | 7.03 | 15.525 | 20.35 | 23.025 | 23.97 | 25.5 | 0.95 | 2.56 |
| Ca T | 0.77 | DS | <0.05 | 46 | 0 | 10.35 | 8.94 | 86.34 | 2.985 | 2.65 | 4.3825 | 6.835 | 14.975 | 21.15 | 22.75 | 28.09 | 49.6 | 2.23 | 10.01 |
| Ca T | 0.77 | RS | <0.05 | 46 | 0 | 10.08 | 6.95 | 68.92 | 3.58 | 2.62 | 4.395 | 7.56 | 16.1 | 20.85 | 23.4 | 24.42 | 25.5 | 0.89 | 2.41 |
| TOC | <0.05 | DS | <0.05 | 46 | 0 | 3.24 | 2.17 | 67.02 | 0.5 | 1.5 | 2 | 2.4 | 3.2 | 5.2 | 8.6 | 10.18 | 10.9 | 2.32 | 8.35 |
| TOC | <0.05 | RS | <0.05 | 46 | 0 | 5.86 | 3.93 | 67.03 | 1.3 | 1.9 | 3.1 | 3.8 | 9.3 | 12.5 | 13.375 | 13.62 | 13.8 | 0.90 | 2.22 |
| Cl | 0.11 | DS | <0.05 | 46 | 10.87 | 13.15 | 28.38 | 215.79 | 2.995 | <1 | 1.9525 | 4.14 | 10.448 | 27.75 | 54.8 | 73.68 | 177 | 4.69 | 28.44 |
| Cl | 0.11 | RS | <0.05 | 46 | 6.52 | 5.57 | 8.20 | 147.22 | 1.145 | <1 | 1.7475 | 2.515 | 3.958 | 12.05 | 26.2 | 34.11 | 36.9 | 2.89 | 11.11 |
| Cu T | <0.05 | DS | <0.05 | 46 | 78.26 | 0.002 | 0.005 | 262.79 | 0 | <0.001 | <0.001 | <0.001 | 0.0005 | 0.0035 | 0.0075 | 0.0139 | 0.031 | 5.09 | 31.72 |
| Cu T | <0.05 | RS | <0.05 | 46 | 36.96 | 0.003 | 0.004 | 121.92 | 0.0015 | <0.001 | <0.001 | 0.002 | 0.004 | 0.0075 | 0.012 | 0.018 | 0.018 | 2.19 | 7.90 |
| EC | 0.97 | DS | <0.05 | 46 | 0 | 156.63 | 186.79 | 119.26 | 41 | 38 | 53.5 | 91 | 160 | 321 | 561 | 623.2 | 1021 | 2.97 | 13.20 |
| EC | 0.97 | RS | <0.05 | 46 | 0 | 119.33 | 93.72 | 78.54 | 21.5 | 38 | 65.25 | 83 | 136.25 | 224.5 | 349 | 413 | 431 | 2.09 | 7.09 |
| Cr D | <0.05 | DS | <0.05 | 46 | 63.04 | 0.0008 | 0.0006 | 69.04 | 0 | <0.001 | <0.001 | <0.001 | 0.001 | 0.002 | 0.002 | 0.0021 | 0.003 | 2.04 | 6.94 |
| Cr D | <0.05 | RS | <0.05 | 46 | 34.78 | 0.0013 | 0.001 | 75.58 | 0.0005 | <0.001 | <0.001 | 0.001 | 0.002 | 0.002 | 0.003 | 0.004 | 0.005 | 2.02 | 8.09 |
| Cr T | <0.05 | DS | <0.05 | 46 | 52.17 | 0.0012 | 0.0012 | 93.47 | 0 | <0.001 | <0.001 | <0.001 | 0.002 | 0.002 | 0.004 | 0.004 | 0.006 | 2.24 | 8.96 |
| Cr T | <0.05 | RS | <0.05 | 46 | 15.22 | 0.005 | 0.008 | 140.72 | 0.0015 | <0.001 | 0.001 | 0.002 | 0.005 | 0.0165 | 0.0245 | 0.0291 | 0.03 | 2.13 | 6.63 |
| Sr D | 0.92 | DS | <0.05 | 46 | 0 | 0.11 | 0.16 | 148.42 | 0.022 | 0.015 | 0.057 | 0.0705 | 0.120 | 0.169 | 0.18575 | 0.292 | 1.13 | 5.96 | 41.40 |
| Sr D | 0.92 | RS | <0.05 | 46 | 0 | 0.09 | 0.05 | 59.33 | 0.0315 | 0.018 | 0.047 | 0.0775 | 0.1085 | 0.1735 | 0.18775 | 0.193 | 0.226 | 0.94 | 3.17 |
| Sr T | 0.96 | DS | <0.05 | 46 | 0 | 0.11 | 0.17 | 145.71 | 0.025 | 0.031 | 0.059 | 0.0745 | 0.122 | 0.177 | 0.196 | 0.341 | 1.17 | 5.90 | 40.77 |
| Sr T | 0.96 | RS | <0.05 | 46 | 0 | 0.09 | 0.05 | 58.69 | 0.032 | 0.02 | 0.05 | 0.0795 | 0.1205 | 0.186 | 0.196 | 0.205 | 0.238 | 0.99 | 3.22 |
| Fe D | <0.05 | DS | <0.05 | 46 | 2.17 | 0.53 | 0.40 | 75.95 | 0.135 | <0.1 | 0.3325 | 0.46 | 0.6275 | 0.805 | 0.945 | 1.392 | 2.67 | 3.67 | 21.51 |
| Fe D | <0.05 | RS | 0.26 | 46 | 8.70 | 0.80 | 0.49 | 60.72 | 0.345 | <0.1 | 0.4325 | 0.77 | 1.098 | 1.425 | 1.553 | 1.786 | 2.11 | 0.43 | 2.87 |
| Fe³⁺ | <0.05 | DS | <0.05 | 46 | 0 | 1.66 | 1.86 | 112.22 | 0.435 | 0.58 | 0.86 | 1.29 | 1.725 | 2.400 | 3.058 | 4.137 | 13.2 | 5.49 | 37.08 |
| Fe³⁺ | <0.05 | RS | <0.05 | 46 | 0 | 3.31 | 2.73 | 82.49 | 0.825 | 0.32 | 1.905 | 2.58 | 3.563 | 4.955 | 8.96 | 12.83 | 14 | 2.57 | 10.30 |
| Fe²⁺ | <0.05 | DS | <0.05 | 46 | 26.09 | 0.22 | 0.43 | 195.31 | 0.08 | <0.05 | 0.034 | 0.12 | 0.195 | 0.32 | 0.7775 | 1.157 | 2.75 | 5.03 | 31.54 |
| Fe²⁺ | <0.05 | RS | <0.05 | 46 | 10.87 | 0.27 | 0.20 | 75.10 | 0.17 | <0.05 | 0.1 | 0.28 | 0.395 | 0.5 | 0.55 | 0.667 | 1 | 1.11 | 5.23 |
| Fe T | <0.05 | DS | <0.05 | 46 | 0 | 1.88 | 2.22 | 118.22 | 0.38 | 0.58 | 1.15 | 1.56 | 1.898 | 2.590 | 3.238 | 4.605 | 15.9 | 5.80 | 39.94 |
| Fe T | <0.05 | RS | <0.05 | 46 | 0 | 3.58 | 2.71 | 75.70 | 0.97 | 0.56 | 2.0875 | 2.94 | 4.015 | 5.61 | 9.05 | 12.84 | 14.1 | 2.44 | 9.78 |
| P D | 0.06 | DS | <0.05 | 46 | 26.09 | 0.18 | 0.60 | 331.09 | 0.03 | <0.02 | 0.0125 | 0.04 | 0.1 | 0.27 | 0.58 | 1.255 | 4 | 6.01 | 41.25 |
| P D | 0.06 | RS | <0.05 | 46 | 2.17 | 0.12 | 0.23 | 195.58 | 0.03 | <0.02 | 0.03 | 0.06 | 0.0975 | 0.195 | 0.37 | 0.528 | 1.5 | 5.38 | 35.29 |
| P T | 0.07 | DS | <0.05 | 46 | 0 | 0.29 | 0.78 | 273.10 | 0.04 | 0.02 | 0.03 | 0.07 | 0.17 | 0.435 | 1.335 | 2.322 | 4.86 | 4.95 | 29.90 |
| P T | 0.07 | RS | <0.05 | 46 | 0 | 0.19 | 0.26 | 140.64 | 0.06 | 0.03 | 0.06 | 0.11 | 0.2325 | 0.355 | 0.4775 | 0.684 | 1.71 | 4.50 | 27.87 |
| Li T | 0.56 | DS | <0.05 | 46 | 47.83 | 0.003 | 0.009 | 344.36 | 0.0005 | <0.001 | <0.001 | 0.001 | 0.002 | 0.002 | 0.006 | 0.016 | 0.061 | 6.39 | 45.14 |
| Li T | 0.56 | RS | <0.05 | 46 | 58.70 | 0.001 | 0.002 | 115.69 | 0 | <0.001 | <0.001 | <0.001 | 0.002 | 0.003 | 0.005 | 0.005 | 0.008 | 2.71 | 11.35 |
| Mg D | 0.08 | DS | <0.05 | 46 | 0 | 2.20 | 1.21 | 55.02 | 0.49 | 0.83 | 1.3675 | 2.125 | 2.475 | 3.065 | 3.79 | 4.458 | 8.49 | 3.27 | 18.97 |
| Mg D | 0.08 | RS | 0.09 | 46 | 0 | 1.82 | 0.59 | 32.26 | 0.43 | 0.95 | 1.39 | 1.745 | 2.193 | 2.685 | 2.833 | 3.133 | 3.34 | 0.61 | 2.92 |
| Mg T | 0.08 | DS | <0.05 | 46 | 0 | 2.33 | 1.23 | 53.00 | 0.61 | 1.06 | 1.455 | 2.23 | 2.753 | 3.370 | 3.888 | 4.522 | 8.59 | 2.99 | 17.13 |
| Mg T | 0.08 | RS | 0.30 | 46 | 0 | 1.93 | 0.60 | 31.30 | 0.485 | 0.95 | 1.43 | 1.9 | 2.378 | 2.775 | 2.93 | 3.234 | 3.36 | 0.41 | 2.59 |
| Mn D | 0.16 | DS | <0.05 | 46 | 4.35 | 0.09 | 0.09 | 98.27 | 0.03 | <0.02 | 0.0325 | 0.06 | 0.0975 | 0.17 | 0.255 | 0.352 | 0.46 | 2.57 | 10.83 |
| Mn D | 0.16 | RS | <0.05 | 46 | 8.70 | 0.07 | 0.08 | 113.65 | 0.02 | <0.02 | 0.03 | 0.05 | 0.0675 | 0.115 | 0.1475 | 0.204 | 0.51 | 4.63 | 29.17 |
| Mn T | 0.16 | DS | <0.05 | 46 | 2.17 | 0.12 | 0.11 | 94.44 | 0.045 | <0.02 | 0.0425 | 0.085 | 0.14 | 0.21 | 0.42 | 0.461 | 0.47 | 2.01 | 6.69 |
| Mn T | 0.16 | RS | <0.05 | 46 | 0 | 0.21 | 0.33 | 154.88 | 0.03 | 0.03 | 0.0625 | 0.09 | 0.1575 | 0.45 | 0.9875 | 1.396 | 1.45 | 2.93 | 10.97 |
| Ni D | <0.05 | DS | <0.05 | 46 | 19.57 | 0.010 | 0.009 | 92.07 | 0.0045 | <0.002 | 0.004 | 0.0095 | 0.014 | 0.019 | 0.024 | 0.028 | 0.053 | 2.38 | 12.21 |
| Ni D | <0.05 | RS | 0.37 | 46 | 2.17 | 0.018 | 0.010 | 56.53 | 0.0075 | <0.002 | 0.011 | 0.017 | 0.025 | 0.0315 | 0.035 | 0.040 | 0.044 | 0.39 | 2.67 |
| Ni T | <0.05 | DS | <0.05 | 46 | 0 | 0.04 | 0.05 | 125.45 | 0.0095 | 0.003 | 0.019 | 0.0285 | 0.038 | 0.0545 | 0.0575 | 0.096 | 0.321 | 5.77 | 39.66 |
| Ni T | <0.05 | RS | <0.05 | 46 | 0 | 0.08 | 0.06 | 76.83 | 0.0195 | 0.016 | 0.046 | 0.07 | 0.083 | 0.138 | 0.202 | 0.280 | 0.337 | 2.50 | 10.29 |
| NO₃ | 0.23 | DS | <0.05 | 46 | 6.52 | 1.09 | 1.30 | 119.75 | 0.495 | <0.02 | 0.155 | 0.525 | 1.705 | 2.3 | 3.425 | 4.418 | 6.38 | 2.00 | 8.20 |
| NO₃ | 0.23 | RS | <0.05 | 46 | 2.17 | 0.86 | 2.12 | 246.31 | 0.305 | <0.02 | 0.13 | 0.425 | 0.805 | 1.475 | 1.963 | 3.744 | 14.4 | 6.03 | 41.89 |
| NO₂ | 0.05 | DS | <0.05 | 46 | 19.57 | 0.12 | 0.18 | 149.29 | 0.035 | <0.01 | 0.02 | 0.04 | 0.16 | 0.26 | 0.595 | 0.68 | 0.77 | 2.27 | 7.81 |
| NO₂ | 0.05 | RS | <0.05 | 46 | 39.13 | 0.06 | 0.08 | 128.28 | 0.02 | <0.01 | <0.01 | 0.025 | 0.1025 | 0.16 | 0.2175 | 0.272 | 0.29 | 1.56 | 4.64 |
| N T | 0.81 | DS | <0.05 | 46 | 30.43 | 2.48 | 2.89 | 116.59 | 0.9 | <0.3 | <0.3 | 1.05 | 4.325 | 6.55 | 7.525 | 8.19 | 12.6 | 1.40 | 4.88 |
| N T | 0.81 | RS | <0.05 | 46 | 4.35 | 1.73 | 1.98 | 114.68 | 0.6 | <0.3 | 0.5 | 1 | 1.95 | 4.05 | 6.125 | 8.06 | 8.6 | 2.15 | 7.34 |
| DO | 0.06 | DS | 0.06 | 46 | 8.70 | 6.18 | 2.86 | 46.31 | 2.505 | <2 | 3.84 | 6.19 | 8.745 | 9.905 | 10.25 | 10.61 | 10.7 | -0.17 | 2.02 |
| DO | 0.06 | RS | <0.05 | 46 | 6.52 | 5.12 | 1.54 | 30.12 | 0.725 | <2 | 4.7225 | 5.41 | 6.103 | 6.615 | 7.17 | 7.492 | 7.6 | -1.22 | 4.71 |
| pH | 0.23 | DS | 0.19 | 46 | 0 | 6.75 | 0.69 | 10.18 | 0.495 | 5.28 | 6.275 | 6.875 | 7.268 | 7.535 | 7.663 | 7.763 | 8.06 | -0.40 | 2.34 |
| pH | 0.23 | RS | 0.09 | 46 | 0 | 6.99 | 0.33 | 4.78 | 0.175 | 6.26 | 6.81 | 6.935 | 7.19 | 7.370 | 7.483 | 7.790 | 8.06 | 0.75 | 4.62 |
| K D | 0.22 | DS | <0.05 | 46 | 0 | 3.43 | 3.33 | 97.29 | 0.575 | 0.69 | 1.7275 | 2.205 | 3.115 | 6.64 | 12.125 | 14.52 | 14.7 | 2.41 | 8.31 |
| K D | 0.22 | RS | <0.05 | 46 | 0 | 2.95 | 1.49 | 50.45 | 0.555 | 1.27 | 2.06 | 2.595 | 3.203 | 4.230 | 5.678 | 7.899 | 8.79 | 2.33 | 9.40 |
| K T | 0.26 | DS | <0.05 | 46 | 0 | 3.73 | 3.60 | 96.36 | 0.61 | 0.94 | 1.855 | 2.335 | 3.695 | 7.485 | 12.775 | 15.3 | 16.2 | 2.36 | 8.03 |
| K T | 0.26 | RS | <0.05 | 46 | 0 | 3.15 | 1.59 | 50.45 | 0.65 | 1.39 | 2.1825 | 2.81 | 3.56 | 4.720 | 6.168 | 8.663 | 8.96 | 2.22 | 8.66 |
| Eh | <0.05 | DS | <0.05 | 46 | 0 | 48.90 | 23.89 | 48.85 | 20.15 | 6 | 28.55 | 47 | 68.075 | 82.4 | 87.3 | 89.96 | 91.4 | 0.22 | 1.86 |
| Eh | <0.05 | RS | <0.05 | 46 | 0 | 21.33 | 12.95 | 60.74 | 5.95 | 1 | 14.425 | 18.8 | 26.6 | 38.7 | 46.65 | 56.35 | 57.7 | 1.08 | 4.13 |
| SiO₂ | 0.25 | DS | 0.16 | 46 | 0 | 16.12 | 6.18 | 38.32 | 3.95 | 5.24 | 10.925 | 15.8 | 19.2 | 24.9 | 27.35 | 28.76 | 30.2 | 0.47 | 2.62 |
| SiO₂ | 0.25 | RS | 0.05 | 46 | 0 | 17.61 | 6.19 | 35.15 | 5.3 | 6.71 | 12.125 | 17.55 | 21.5 | 26.7 | 28.025 | 28.42 | 28.6 | 0.24 | 2.02 |
| Na D | <0.05 | DS | <0.05 | 46 | 0 | 14.13 | 21.32 | 150.87 | 3.44 | 2.23 | 5.69 | 8.34 | 12.175 | 23.15 | 44.825 | 57.99 | 138 | 4.74 | 29.11 |
| Na D | <0.05 | RS | <0.05 | 46 | 0 | 7.51 | 6.71 | 89.34 | 1.46 | 2.07 | 4.075 | 5.475 | 7.173 | 13.15 | 24.05 | 27.66 | 34.5 | 2.61 | 9.93 |
| Na T | <0.05 | DS | <0.05 | 46 | 0 | 15.96 | 24.69 | 154.69 | 3.325 | 3.25 | 5.9125 | 9.015 | 14.675 | 26.95 | 47.15 | 61.03 | 163 | 5.01 | 31.94 |
| Na T | <0.05 | RS | <0.05 | 46 | 0 | 7.83 | 6.74 | 85.99 | 1.72 | 2.18 | 4.1525 | 5.76 | 7.703 | 13.3 | 24.075 | 28.35 | 35.1 | 2.62 | 10.07 |
| TDS | 0.10 | DS | <0.05 | 46 | 0 | 108.07 | 89.72 | 83.03 | 22.25 | 31.1 | 65.25 | 81.5 | 120.75 | 186.5 | 247 | 298 | 586 | 3.73 | 20.83 |
| TDS | 0.10 | RS | <0.05 | 46 | 0 | 109.08 | 47.34 | 43.40 | 24 | 38 | 75.5 | 97.35 | 130.75 | 161.5 | 181.75 | 238.2 | 267 | 1.23 | 5.08 |
| TSS | <0.05 | DS | <0.05 | 46 | 10.87 | 30.65 | 44.45 | 145.05 | 10.1 | <7 | 11.375 | 20.25 | 31.625 | 42 | 86.875 | 162.4 | 274 | 4.28 | 24.06 |
| TSS | <0.05 | RS | <0.05 | 46 | 6.52 | 117.91 | 185.38 | 157.22 | 29 | <7 | 24.25 | 45.35 | 115.25 | 315 | 479.75 | 593.5 | 976 | 2.94 | 13.08 |
| ST | <0.05 | DS | <0.05 | 46 | 0 | 138.78 | 105.09 | 75.72 | 26 | 37.1 | 81 | 98.5 | 150 | 258 | 333.5 | 386.7 | 618 | 2.70 | 12.15 |
| ST | <0.05 | RS | <0.05 | 46 | 0 | 227.89 | 182.53 | 80.10 | 60.5 | 58 | 117.25 | 170.5 | 274 | 477 | 552.5 | 669.8 | 1046 | 2.58 | 11.42 |
| SO₄²⁻ | 0.06 | DS | <0.05 | 46 | 36.96 | 5.86 | 17.82 | 304.24 | 1.44 | <1 | <1 | 1.94 | 6.01 | 7.570 | 11.253 | 24.350 | 122 | 6.42 | 45.57 |
| SO₄²⁻ | 0.06 | RS | <0.05 | 46 | 58.70 | 3.00 | 6.37 | 212.23 | 0 | <1 | <1 | <1 | 2.99 | 7.010 | 9.563 | 13.850 | 41.3 | 5.12 | 33.16 |
| T | <0.05 | DS | <0.05 | 46 | 0 | 19.42 | 2.64 | 13.59 | 2 | 15.5 | 17.15 | 19 | 21.225 | 23.75 | 23.875 | 25.21 | 25.3 | 0.57 | 2.47 |
| T | <0.05 | RS | 0.15 | 46 | 0 | 24.69 | 1.60 | 6.46 | 1.15 | 21.9 | 23.45 | 24.5 | 25.675 | 27 | 27.375 | 27.8 | 28.7 | 0.55 | 2.63 |
| Ti D | 0.22 | DS | <0.05 | 46 | 47.83 | 0.001 | 0.001 | 84.28 | 0.0005 | <0.001 | <0.001 | 0.001 | 0.002 | 0.003 | 0.004 | 0.0041 | 0.005 | 1.24 | 3.75 |
| Ti D | 0.22 | RS | <0.05 | 46 | 34.78 | 0.002 | 0.003 | 128.86 | 0.0005 | <0.001 | <0.001 | 0.001 | 0.002 | 0.006 | 0.008 | 0.011 | 0.015 | 2.76 | 11.39 |
| Ti T | <0.05 | DS | <0.05 | 46 | 23.91 | 0.005 | 0.006 | 138.02 | 0.002 | <0.001 | 0.001 | 0.0025 | 0.006 | 0.010 | 0.013 | 0.030 | 0.03 | 3.00 | 13.04 |
| Ti T | <0.05 | RS | <0.05 | 46 | 13.04 | 0.024 | 0.046 | 192.82 | 0.00425 | <0.001 | 0.002 | 0.005 | 0.022 | 0.0495 | 0.1265 | 0.18 | 0.225 | 3.07 | 12.74 |
| Turbidity | <0.05 | DS | <0.05 | 46 | 0 | 24.28 | 35.95 | 148.06 | 4.5 | 7.34 | 10.7 | 15 | 21.075 | 34.2 | 64.45 | 109.56 | 237 | 5.02 | 31.39 |
| Turbidity | <0.05 | RS | <0.05 | 46 | 0 | 109.31 | 121.90 | 111.51 | 39.3 | 15.1 | 38.4 | 70.05 | 126.5 | 233.5 | 311.75 | 410 | 698 | 3.00 | 14.69 |
| V T | <0.05 | DS | <0.05 | 46 | 67.39 | 0.001 | 0.001 | 108.54 | 0 | <0.001 | <0.001 | <0.001 | 0.001 | 0.001 | 0.003 | 0.003 | 0.006 | 4.07 | 22.01 |
| V T | <0.05 | RS | <0.05 | 46 | 26.09 | 0.005 | 0.007 | 155.23 | 0.0015 | <0.001 | 0.001 | 0.002 | 0.005 | 0.011 | 0.021 | 0.030 | 0.033 | 2.70 | 10.34 |

## MVS-MLU

| **Parameter** | **pMW** | **Dataset** | **pSW** | **n** | **% <LD** | **µ** | **s** | **% CV** | **MAD** | **Min** | **P25** | **P50** | **P75** | **P90** | **P95** | **P98** | **Max** | **skewness** | **kurtosis** |
| --- | --- | --- | --- | --- | --- | --- | --- | --- | --- | --- | --- | --- | --- | --- | --- | --- | --- | --- | --- |
| Alk HCO₃ | 0.35 | DS | <0.05 | 58 | 0 | 29.83 | 23.12 | 77.49 | 6.25 | 7.29 | 19.7 | 24.2 | 31.025 | 45.69 | 78.49 | 113.638 | 128 | 2.80 | 11.63 |
| Alk HCO₃ | 0.35 | RS | <0.05 | 58 | 0 | 24.27 | 12.60 | 51.92 | 7.8 | 4.35 | 15.4 | 23.45 | 27.75 | 34.69 | 51.54 | 59.866 | 70.5 | 1.57 | 6.35 |
| Alk T | 0.35 | DS | <0.05 | 58 | 0 | 29.83 | 23.12 | 77.49 | 6.25 | 7.29 | 19.7 | 24.2 | 31.025 | 45.69 | 78.49 | 113.638 | 128 | 2.80 | 11.63 |
| Alk T | 0.35 | RS | <0.05 | 58 | 0 | 24.27 | 12.60 | 51.92 | 7.8 | 4.35 | 15.4 | 23.45 | 27.75 | 34.69 | 51.54 | 59.866 | 70.5 | 1.57 | 6.35 |
| Al D | <0.05 | DS | <0.05 | 58 | 46.55 | 0.04 | 0.05 | 123.98 | 0.01 | <0.02 | <0.02 | 0.02 | 0.05 | 0.083 | 0.1215 | 0.139 | 0.3 | 3.33 | 18.13 |
| Al D | <0.05 | RS | <0.05 | 58 | 24.14 | 0.10 | 0.10 | 97.98 | 0.06 | <0.02 | 0.02 | 0.07 | 0.15 | 0.213 | 0.2535 | 0.339 | 0.46 | 1.55 | 5.98 |
| Al T | <0.05 | DS | <0.05 | 58 | 6.90 | 0.13 | 0.13 | 98.56 | 0.055 | <0.02 | 0.04 | 0.085 | 0.19 | 0.316 | 0.343 | 0.4374 | 0.67 | 1.85 | 7.37 |
| Al T | <0.05 | RS | <0.05 | 58 | 1.72 | 0.80 | 1.29 | 161.83 | 0.1 | <0.02 | 0.16 | 0.215 | 0.4325 | 2.661 | 3.564 | 5.110 | 5.51 | 2.31 | 7.80 |
| Ba D | 0.32 | DS | <0.05 | 58 | 0 | 0.026 | 0.014 | 55.28 | 0.007 | 0.005 | 0.015 | 0.022 | 0.032 | 0.0456 | 0.0556 | 0.059 | 0.068 | 1.11 | 3.88 |
| Ba D | 0.32 | RS | <0.05 | 58 | 1.72 | 0.023 | 0.013 | 56.45 | 0.006 | <0.001 | 0.0145 | 0.02 | 0.026 | 0.0406 | 0.0469 | 0.054 | 0.071 | 1.39 | 5.45 |
| Ba T | 0.73 | DS | <0.05 | 58 | 0 | 0.030 | 0.018 | 60.93 | 0.009 | 0.006 | 0.017 | 0.026 | 0.037 | 0.0533 | 0.0606 | 0.067 | 0.106 | 1.72 | 7.59 |
| Ba T | 0.73 | RS | <0.05 | 58 | 0 | 0.031 | 0.018 | 57.70 | 0.0085 | 0.005 | 0.019 | 0.025 | 0.038 | 0.0553 | 0.0655 | 0.077 | 0.08 | 1.16 | 3.86 |
| Ca D | 0.83 | DS | <0.05 | 58 | 0 | 4.89 | 3.83 | 78.43 | 1.325 | 1.06 | 2.615 | 3.69 | 5.755 | 8.711 | 13.935 | 16.83 | 19 | 2.10 | 7.66 |
| Ca D | 0.83 | RS | <0.05 | 58 | 0 | 4.84 | 3.46 | 71.50 | 1.725 | 0.74 | 2.6825 | 3.67 | 5.805 | 8.626 | 11.52 | 15.858 | 16.1 | 1.78 | 6.44 |
| Ca T | 0.78 | DS | <0.05 | 58 | 0 | 5.27 | 4.44 | 84.31 | 1.37 | 1.17 | 2.7225 | 3.71 | 6.1 | 9.344 | 14.375 | 19.338 | 24 | 2.40 | 9.58 |
| Ca T | 0.78 | RS | <0.05 | 58 | 0 | 5.15 | 3.76 | 72.96 | 1.785 | 0.81 | 2.8725 | 3.76 | 6.048 | 9.101 | 12.26 | 16.516 | 19.1 | 1.92 | 7.14 |
| TOC | 0.25 | DS | <0.05 | 58 | 0 | 1.83 | 1.34 | 73.22 | 0.3 | 0.6 | 1.2 | 1.5 | 1.875 | 2.86 | 5.515 | 5.772 | 7.8 | 2.80 | 11.53 |
| TOC | 0.25 | RS | <0.05 | 58 | 6.90 | 1.90 | 1.21 | 63.60 | 0.55 | <0.5 | 1.2 | 1.85 | 2.3 | 3.33 | 3.545 | 5.348 | 6.7 | 1.56 | 7.28 |
| Cl | 0.12 | DS | <0.05 | 58 | 17.24 | 4.17 | 6.84 | 164.08 | 0.965 | <1 | 1.3475 | 1.89 | 3.243 | 7.647 | 17.88 | 29.438 | 35.3 | 3.29 | 14.03 |
| Cl | 0.12 | RS | <0.05 | 58 | 20.69 | 2.55 | 3.35 | 131.29 | 0.565 | <1 | 1.33 | 1.53 | 2.355 | 3.489 | 11.98 | 14.29 | 16.6 | 3.09 | 12.15 |
| Cu T | <0.05 | DS | <0.05 | 58 | 75.86 | 0.001 | 0.001 | 97.95 | 0 | <0.001 | <0.001 | <0.001 | 0.0005 | 0.001 | 0.002 | 0.003 | 0.005 | 3.57 | 17.54 |
| Cu T | <0.05 | RS | <0.05 | 58 | 55.17 | 0.001 | 0.002 | 105.57 | 0 | <0.001 | <0.001 | <0.001 | 0.002 | 0.003 | 0.004 | 0.006 | 0.008 | 2.14 | 8.42 |
| EC | 0.31 | DS | <0.05 | 58 | 0 | 70.28 | 68.54 | 97.53 | 16 | 17 | 37 | 49.5 | 69.5 | 94.2 | 259.15 | 311.86 | 328 | 2.85 | 10.78 |
| EC | 0.31 | RS | <0.05 | 58 | 0 | 67.91 | 46.28 | 68.14 | 17 | 11 | 40.25 | 60 | 73 | 94.3 | 173 | 221.82 | 246 | 2.31 | 8.83 |
| Cr D | 0.49 | DS | <0.05 | 58 | 74.14 | 0.0007 | 0.0005 | 66.89 | 0 | <0.001 | <0.001 | <0.001 | 0.001 | 0.001 | 0.002 | 0.002 | 0.003 | 3.00 | 13.05 |
| Cr D | 0.49 | RS | <0.05 | 58 | 67.24 | 0.0007 | 0.0004 | 52.54 | 0 | <0.001 | <0.001 | <0.001 | 0.001 | 0.001 | 0.001 | 0.002 | 0.002 | 2.17 | 7.99 |
| Cr T | <0.05 | DS | <0.05 | 58 | 50.00 | 0.001 | 0.002 | 139.73 | 0.00025 | <0.001 | <0.001 | 0.0008 | 0.002 | 0.0023 | 0.003 | 0.0056 | 0.014 | 5.02 | 33.49 |
| Cr T | <0.05 | RS | <0.05 | 58 | 34.48 | 0.003 | 0.003 | 120.20 | 0.0015 | <0.001 | <0.001 | 0.002 | 0.003 | 0.006 | 0.0103 | 0.01372 | 0.016 | 2.30 | 8.63 |
| Sr D | 0.30 | DS | <0.05 | 58 | 0 | 0.038 | 0.036 | 95.31 | 0.015 | 0.005 | 0.016 | 0.028 | 0.047 | 0.0591 | 0.08075 | 0.161 | 0.217 | 3.13 | 15.16 |
| Sr D | 0.30 | RS | <0.05 | 58 | 0 | 0.033 | 0.030 | 88.85 | 0.0115 | 0.005 | 0.017 | 0.023 | 0.042 | 0.054 | 0.083 | 0.128 | 0.163 | 2.65 | 11.20 |
| Sr T | 0.47 | DS | <0.05 | 58 | 0 | 0.040 | 0.037 | 92.57 | 0.016 | 0.007 | 0.017 | 0.03 | 0.05 | 0.060 | 0.083 | 0.163 | 0.222 | 3.06 | 14.89 |
| Sr T | 0.47 | RS | <0.05 | 58 | 0 | 0.036 | 0.032 | 89.17 | 0.014 | 0.005 | 0.018 | 0.025 | 0.044 | 0.0582 | 0.0852 | 0.157 | 0.167 | 2.66 | 11.23 |
| Fe D | <0.05 | DS | <0.05 | 58 | 10.34 | 0.38 | 0.23 | 62.14 | 0.135 | <0.1 | 0.2125 | 0.335 | 0.4775 | 0.656 | 0.825 | 0.91 | 1.14 | 0.99 | 4.26 |
| Fe D | <0.05 | RS | 0.60 | 58 | 1.72 | 0.63 | 0.29 | 45.56 | 0.195 | <0.1 | 0.4125 | 0.67 | 0.81 | 0.943 | 1.088 | 1.147 | 1.45 | 0.17 | 3.20 |
| Fe³⁺ | <0.05 | DS | <0.05 | 58 | 3.45 | 0.91 | 0.64 | 70.43 | 0.3 | <0.05 | 0.5225 | 0.735 | 1.138 | 1.456 | 2.342 | 2.668 | 3.59 | 1.96 | 8.43 |
| Fe³⁺ | <0.05 | RS | <0.05 | 58 | 0 | 1.96 | 1.45 | 73.74 | 0.635 | 0.21 | 1.0075 | 1.44 | 2.6175 | 3.993 | 4.648 | 5.176 | 7.48 | 1.52 | 5.75 |
| Fe²⁺ | <0.05 | DS | <0.05 | 58 | 41.38 | 0.15 | 0.25 | 164.13 | 0.045 | <0.05 | <0.05 | 0.07 | 0.14 | 0.341 | 0.737 | 0.89 | 1.35 | 3.10 | 13.16 |
| Fe²⁺ | <0.05 | RS | <0.05 | 58 | 17.24 | 0.17 | 0.11 | 66.51 | 0.075 | <0.05 | 0.0825 | 0.155 | 0.23 | 0.312 | 0.37 | 0.396 | 0.52 | 0.74 | 3.46 |
| Fe T | <0.05 | DS | <0.05 | 58 | 0 | 1.05 | 0.80 | 75.58 | 0.255 | 0.12 | 0.6075 | 0.83 | 1.21 | 1.735 | 2.914 | 3.506 | 4.05 | 2.08 | 7.65 |
| Fe T | <0.05 | RS | <0.05 | 58 | 0 | 2.13 | 1.47 | 69.22 | 0.72 | 0.22 | 1.1525 | 1.695 | 2.923 | 4.267 | 4.995 | 5.319 | 7.59 | 1.39 | 5.32 |
| P D | <0.05 | DS | <0.05 | 58 | 68.97 | 0.06 | 0.16 | 286.38 | 0 | <0.02 | <0.02 | <0.02 | 0.02 | 0.081 | 0.193 | 0.7412 | 0.93 | 4.52 | 23.71 |
| P D | <0.05 | RS | <0.05 | 58 | 36.21 | 0.04 | 0.06 | 127.39 | 0.02 | <0.02 | <0.02 | 0.03 | 0.0475 | 0.089 | 0.1745 | 0.226 | 0.29 | 2.77 | 11.14 |
| P T | <0.05 | DS | <0.05 | 58 | 43.10 | 0.09 | 0.23 | 253.34 | 0.01 | <0.02 | <0.02 | 0.02 | 0.06 | 0.16 | 0.242 | 0.9186 | 1.4 | 4.72 | 26.64 |
| P T | <0.05 | RS | <0.05 | 58 | 15.52 | 0.08 | 0.09 | 108.14 | 0.03 | <0.02 | 0.03 | 0.05 | 0.0975 | 0.2 | 0.2705 | 0.339 | 0.39 | 1.93 | 6.44 |
| Li T | 0.38 | DS | <0.05 | 58 | 46.55 | 0.001 | 0.001 | 85.58 | 0.0005 | <0.001 | <0.001 | 0.001 | 0.002 | 0.003 | 0.003 | 0.005 | 0.006 | 1.45 | 5.14 |
| Li T | 0.38 | RS | <0.05 | 58 | 50.00 | 0.001 | 0.001 | 81.95 | 0.00025 | <0.001 | <0.001 | 0.0008 | 0.002 | 0.003 | 0.003 | 0.0039 | 0.004 | 1.40 | 3.96 |
| Mg D | 0.28 | DS | 0.08 | 58 | 0 | 2.11 | 0.98 | 46.49 | 0.695 | 0.39 | 1.4475 | 1.9 | 2.685 | 3.533 | 3.946 | 4.066 | 4.52 | 0.54 | 2.73 |
| Mg D | 0.28 | RS | 0.24 | 58 | 0 | 1.89 | 0.83 | 44.09 | 0.555 | 0.31 | 1.3275 | 1.785 | 2.37 | 3.013 | 3.458 | 3.569 | 3.66 | 0.35 | 2.60 |
| Mg T | 0.28 | DS | 0.07 | 58 | 0 | 2.26 | 1.07 | 47.15 | 0.68 | 0.46 | 1.5475 | 2.065 | 2.838 | 3.995 | 4.302 | 4.489 | 4.88 | 0.57 | 2.82 |
| Mg T | 0.28 | RS | 0.33 | 58 | 0 | 2.02 | 0.88 | 43.75 | 0.545 | 0.32 | 1.4375 | 1.955 | 2.56 | 3.405 | 3.634 | 3.736 | 3.93 | 0.31 | 2.55 |
| Mn D | 0.11 | DS | <0.05 | 58 | 12.07 | 0.13 | 0.17 | 130.30 | 0.04 | <0.02 | 0.04 | 0.07 | 0.12 | 0.289 | 0.463 | 0.5832 | 0.96 | 2.97 | 13.77 |
| Mn D | 0.11 | RS | <0.05 | 58 | 3.45 | 0.14 | 0.13 | 92.63 | 0.05 | <0.02 | 0.06 | 0.09 | 0.17 | 0.33 | 0.473 | 0.5416 | 0.56 | 1.75 | 5.65 |
| Mn T | <0.05 | DS | <0.05 | 58 | 8.62 | 0.16 | 0.18 | 117.14 | 0.05 | <0.02 | 0.055 | 0.1 | 0.165 | 0.355 | 0.505 | 0.6674 | 1.03 | 2.79 | 12.26 |
| Mn T | <0.05 | RS | <0.05 | 58 | 1.72 | 0.21 | 0.18 | 85.52 | 0.085 | <0.02 | 0.1 | 0.165 | 0.29 | 0.386 | 0.605 | 0.7588 | 0.94 | 2.02 | 7.80 |
| Ni D | <0.05 | DS | <0.05 | 58 | 5.17 | 0.010 | 0.006 | 64.39 | 0.0035 | <0.002 | 0.005 | 0.008 | 0.014 | 0.018 | 0.020 | 0.023 | 0.03 | 1.05 | 4.05 |
| Ni D | <0.05 | RS | 0.40 | 58 | 1.72 | 0.016 | 0.007 | 46.94 | 0.0045 | <0.002 | 0.010 | 0.0165 | 0.021 | 0.0236 | 0.029 | 0.029 | 0.038 | 0.30 | 3.45 |
| Ni T | <0.05 | DS | <0.05 | 58 | 0 | 0.02 | 0.01 | 61.71 | 0.0075 | 0.003 | 0.014 | 0.022 | 0.029 | 0.0408 | 0.0543 | 0.068 | 0.075 | 1.54 | 5.84 |
| Ni T | <0.05 | RS | <0.05 | 58 | 0 | 0.05 | 0.03 | 66.21 | 0.0165 | 0.005 | 0.029 | 0.0405 | 0.072 | 0.0953 | 0.1023 | 0.114 | 0.193 | 1.45 | 6.59 |
| NO₃ | 0.10 | DS | <0.05 | 58 | 3.45 | 0.52 | 0.47 | 90.19 | 0.255 | <0.02 | 0.195 | 0.36 | 0.85 | 1.042 | 1.161 | 1.8112 | 2.28 | 1.58 | 6.12 |
| NO₃ | 0.10 | RS | <0.05 | 58 | 3.45 | 0.36 | 0.30 | 84.88 | 0.12 | <0.02 | 0.1725 | 0.26 | 0.4475 | 0.755 | 0.9355 | 1.321 | 1.51 | 2.04 | 7.69 |
| NO₂ | 0.08 | DS | <0.05 | 58 | 34.48 | 0.04 | 0.06 | 139.65 | 0.025 | <0.01 | <0.01 | 0.03 | 0.05 | 0.08 | 0.1105 | 0.187 | 0.38 | 3.99 | 23.35 |
| NO₂ | 0.08 | RS | <0.05 | 58 | 46.55 | 0.06 | 0.17 | 279.85 | 0.005 | <0.01 | <0.01 | 0.01 | 0.0375 | 0.126 | 0.2315 | 0.6442 | 1.12 | 4.96 | 29.59 |
| N T | <0.05 | DS | <0.05 | 58 | 5.17 | 4.20 | 4.28 | 101.90 | 1.2 | <0.3 | 1.825 | 3 | 4.2 | 9.7 | 12.655 | 17.884 | 21.6 | 2.39 | 9.00 |
| N T | <0.05 | RS | <0.05 | 58 | 12.07 | 1.10 | 1.19 | 108.55 | 0.3 | <0.3 | 0.4 | 0.7 | 1.175 | 3.04 | 4.315 | 4.4 | 4.4 | 1.91 | 5.68 |
| DO | <0.05 | DS | <0.05 | 58 | 3.45 | 7.64 | 2.55 | 33.33 | 1.54 | <2 | 6.35 | 8.195 | 9.528 | 9.935 | 10.72 | 11.658 | 11.7 | -0.85 | 3.21 |
| DO | <0.05 | RS | <0.05 | 58 | 0 | 6.06 | 1.99 | 32.90 | 1.055 | 2.33 | 4.945 | 5.78 | 6.965 | 7.827 | 10.715 | 11.23 | 12.9 | 1.17 | 5.45 |
| pH | <0.05 | DS | <0.05 | 58 | 0 | 6.43 | 0.66 | 10.31 | 0.6 | 4.92 | 5.8775 | 6.46 | 7.058 | 7.299 | 7.33 | 7.442 | 7.55 | -0.19 | 1.98 |
| pH | <0.05 | RS | <0.05 | 58 | 0 | 6.86 | 0.39 | 5.73 | 0.285 | 5.47 | 6.635 | 6.88 | 7.213 | 7.283 | 7.408 | 7.459 | 7.48 | -0.86 | 4.49 |
| K D | 0.52 | DS | <0.05 | 58 | 8.62 | 1.62 | 1.49 | 92.30 | 0.45 | <0.6 | 0.8325 | 1.285 | 1.7 | 2.253 | 6.076 | 6.712 | 7.46 | 2.73 | 10.46 |
| K D | 0.52 | RS | <0.05 | 58 | 6.90 | 1.42 | 0.69 | 48.52 | 0.45 | <0.6 | 0.8425 | 1.38 | 1.795 | 2.133 | 2.522 | 3.287 | 3.38 | 0.80 | 4.13 |
| K T | 0.79 | DS | <0.05 | 58 | 6.90 | 1.78 | 1.66 | 92.95 | 0.47 | <0.6 | 0.925 | 1.455 | 1.868 | 2.315 | 6.703 | 7.596 | 7.8 | 2.78 | 10.57 |
| K T | 0.79 | RS | <0.05 | 58 | 5.17 | 1.54 | 0.78 | 50.82 | 0.47 | <0.6 | 0.915 | 1.555 | 1.86 | 2.255 | 3.171 | 3.469 | 4.26 | 1.13 | 5.11 |
| Eh | <0.05 | DS | <0.05 | 58 | 0 | 75.85 | 41.00 | 54.05 | 23.2 | 14.5 | 47.675 | 70.95 | 92.975 | 137.9 | 155 | 172.86 | 184 | 0.81 | 3.32 |
| Eh | <0.05 | RS | <0.05 | 58 | 0 | 55.39 | 40.87 | 73.79 | 32.75 | -17.3 | 18.175 | 55.65 | 78.875 | 103.3 | 113.35 | 146.6 | 199 | 0.96 | 4.50 |
| SiO₂ | 0.85 | DS | <0.05 | 58 | 0 | 10.60 | 5.83 | 55.00 | 5.4 | 1.37 | 4.74 | 11.1 | 16.25 | 17.22 | 18.33 | 19.188 | 25 | 0.10 | 1.97 |
| SiO₂ | 0.85 | RS | 0.31 | 58 | 0 | 10.50 | 3.59 | 34.23 | 2.96 | 3.06 | 7.66 | 10.8 | 13.4 | 14.76 | 15.805 | 16.572 | 17.3 | -0.20 | 2.26 |
| Na D | 0.15 | DS | <0.05 | 58 | 0 | 5.44 | 7.04 | 129.45 | 1.13 | 0.81 | 2.53 | 3.4 | 4.985 | 8.159 | 25.6 | 31.474 | 32.7 | 3.15 | 12.27 |
| Na D | 0.15 | RS | <0.05 | 58 | 0 | 3.67 | 2.84 | 77.32 | 0.885 | 0.67 | 2.105 | 3.08 | 3.948 | 6.094 | 9.929 | 13.266 | 14.1 | 2.24 | 8.24 |
| Na T | 0.29 | DS | <0.05 | 58 | 0 | 5.90 | 7.60 | 128.78 | 1.435 | 0.84 | 2.7575 | 3.665 | 5.47 | 8.298 | 25.84 | 32.73 | 37.9 | 3.17 | 12.67 |
| Na T | 0.29 | RS | <0.05 | 58 | 0 | 4.30 | 3.54 | 82.25 | 1.065 | 0.79 | 2.39 | 3.515 | 4.445 | 9.528 | 13.52 | 14.286 | 17.9 | 2.22 | 7.88 |
| TDS | 0.36 | DS | <0.05 | 58 | 0 | 58.24 | 36.13 | 62.02 | 17.6 | 20 | 35 | 48.9 | 69 | 87.5 | 149.45 | 182.1 | 189 | 2.14 | 8.22 |
| TDS | 0.36 | RS | <0.05 | 58 | 0 | 59.27 | 27.85 | 46.99 | 16.2 | 22 | 39.65 | 53.85 | 72.45 | 89.5 | 105 | 129.74 | 169 | 1.51 | 6.55 |
| TSS | 0.19 | DS | <0.05 | 58 | 20.69 | 18.30 | 14.79 | 80.78 | 7.85 | <7 | 8.425 | 15.2 | 24.25 | 33.45 | 37.225 | 44.95 | 92.5 | 2.38 | 12.96 |
| TSS | 0.19 | RS | <0.05 | 58 | 20.69 | 33.76 | 45.77 | 135.59 | 14.15 | <7 | 8.25 | 17.65 | 41.2 | 81.5 | 100.14 | 117.74 | 294 | 3.61 | 20.88 |
| ST | 0.05 | DS | <0.05 | 58 | 0 | 76.74 | 41.05 | 53.49 | 20.95 | 26 | 45.075 | 71 | 89.375 | 118.5 | 164.8 | 205.1 | 218 | 1.59 | 6.16 |
| ST | 0.05 | RS | <0.05 | 58 | 0 | 93.03 | 52.32 | 56.24 | 31.65 | 30.8 | 58.9 | 83.65 | 121 | 149.6 | 170.65 | 198.92 | 340 | 2.09 | 10.56 |
| SO₄²⁻ | 0.25 | DS | <0.05 | 58 | 48.28 | 2.83 | 3.51 | 124.29 | 0.77 | <1 | <1 | 1.27 | 3.608 | 8.156 | 8.675 | 9.597 | 17.6 | 1.91 | 7.28 |
| SO₄²⁻ | 0.25 | RS | <0.05 | 58 | 51.72 | 1.82 | 2.28 | 125.27 | 0 | <1 | <1 | <1 | 2.025 | 5.511 | 7.225 | 7.491 | 11.3 | 2.32 | 8.41 |
| T | <0.05 | DS | <0.05 | 58 | 0 | 18.34 | 2.46 | 13.44 | 1.2 | 13.6 | 16.825 | 17.8 | 19.2 | 21.85 | 23.375 | 25.004 | 25.4 | 1.05 | 4.28 |
| T | <0.05 | RS | 0.53 | 58 | 0 | 22.95 | 1.82 | 7.92 | 1.35 | 19 | 21.725 | 22.6 | 24.275 | 25.7 | 25.93 | 26.272 | 27.5 | 0.31 | 2.73 |
| Ti D | <0.05 | DS | <0.05 | 58 | 68.97 | 0.001 | 0.001 | 69.42 | 0 | <0.001 | <0.001 | <0.001 | 0.001 | 0.002 | 0.002 | 0.002 | 0.002 | 1.38 | 3.16 |
| Ti D | <0.05 | RS | <0.05 | 58 | 41.38 | 0.001 | 0.001 | 67.02 | 0.0005 | <0.001 | <0.001 | 0.001 | 0.002 | 0.002 | 0.003 | 0.003 | 0.003 | 1.21 | 3.51 |
| Ti T | <0.05 | DS | <0.05 | 58 | 41.38 | 0.002 | 0.002 | 100.70 | 0.001 | <0.001 | <0.001 | 0.0015 | 0.003 | 0.006 | 0.006 | 0.008 | 0.01 | 1.49 | 4.86 |
| Ti T | <0.05 | RS | <0.05 | 58 | 20.69 | 0.009 | 0.016 | 179.66 | 0.002 | <0.001 | 0.001 | 0.0025 | 0.006 | 0.0303 | 0.0393 | 0.048 | 0.095 | 3.18 | 15.62 |
| Turbidity | <0.05 | DS | <0.05 | 58 | 0 | 21.28 | 30.91 | 145.26 | 8.095 | 2.48 | 7.2825 | 13.5 | 26.775 | 35.67 | 42.985 | 74.89 | 225 | 5.32 | 36.92 |
| Turbidity | <0.05 | RS | <0.05 | 58 | 0 | 56.33 | 41.33 | 73.37 | 20.35 | 3.36 | 23.675 | 43.35 | 96.825 | 112.9 | 121.05 | 149.36 | 179 | 0.95 | 3.08 |
| V T | <0.05 | DS | <0.05 | 58 | 89.66 | 0.001 | 0.000 | 57.57 | 0 | <0.001 | <0.001 | <0.001 | 0.0005 | 0.001 | 0.001 | 0.002 | 0.002 | 3.60 | 15.21 |
| V T | <0.05 | RS | <0.05 | 58 | 56.90 | 0.002 | 0.003 | 145.80 | 0 | <0.001 | <0.001 | <0.001 | 0.002 | 0.007 | 0.008 | 0.008 | 0.015 | 2.49 | 9.88 |

## TTG-P

| **Parameter** | **pMW** | **Dataset** | **pSW** | **n** | **% <LD** | **µ** | **s** | **% CV** | **MAD** | **Min** | **P25** | **P50** | **P75** | **P90** | **P95** | **P98** | **Max** | **skewness** | **kurtosis** |
| --- | --- | --- | --- | --- | --- | --- | --- | --- | --- | --- | --- | --- | --- | --- | --- | --- | --- | --- | --- |
| Alk HCO₃ | 0.17 | DS | 0.63 | 68 | 0 | 20.55 | 7.15 | 34.79 | 4.8 | 6.09 | 15.175 | 21.1 | 25.225 | 28.71 | 31.835 | 34.784 | 39.6 | 0.08 | 2.83 |
| Alk HCO₃ | 0.17 | RS | 0.25 | 68 | 2.94 | 18.62 | 7.53 | 40.47 | 4.75 | <4 | 14.2 | 19.7 | 23.9 | 26.62 | 30.72 | 32.528 | 35 | -0.28 | 2.69 |
| Alk T | 0.17 | DS | 0.63 | 68 | 0 | 20.55 | 7.15 | 34.79 | 4.8 | 6.09 | 15.175 | 21.1 | 25.225 | 28.71 | 31.835 | 34.784 | 39.6 | 0.08 | 2.83 |
| Alk T | 0.17 | RS | 0.25 | 68 | 2.94 | 18.62 | 7.53 | 40.47 | 4.75 | <4 | 14.2 | 19.7 | 23.9 | 26.62 | 30.72 | 32.528 | 35 | -0.28 | 2.69 |
| Al D | <0.05 | DS | <0.05 | 68 | 44.12 | 0.03 | 0.02 | 81.80 | 0.01 | <0.02 | <0.02 | 0.02 | 0.04 | 0.06 | 0.07 | 0.0832 | 0.13 | 1.68 | 6.90 |
| Al D | <0.05 | RS | <0.05 | 68 | 25.00 | 0.11 | 0.17 | 146.77 | 0.05 | <0.02 | 0.0175 | 0.06 | 0.15 | 0.236 | 0.2765 | 0.4466 | 1.2 | 4.54 | 30.51 |
| Al T | <0.05 | DS | <0.05 | 68 | 4.41 | 0.13 | 0.14 | 102.15 | 0.04 | <0.02 | 0.04 | 0.07 | 0.17 | 0.286 | 0.3925 | 0.4996 | 0.74 | 2.14 | 8.74 |
| Al T | <0.05 | RS | <0.05 | 68 | 0 | 2.56 | 5.35 | 209.51 | 0.585 | 0.03 | 0.22 | 0.7 | 2.288 | 5.105 | 10.325 | 20.816 | 32.8 | 4.10 | 22.03 |
| Ba D | 0.30 | DS | 0.23 | 68 | 0 | 0.021 | 0.007 | 33.79 | 0.005 | 0.002 | 0.016 | 0.022 | 0.027 | 0.0293 | 0.03165 | 0.034 | 0.034 | -0.32 | 2.71 |
| Ba D | 0.30 | RS | <0.05 | 68 | 1.47 | 0.020 | 0.010 | 50.18 | 0.005 | <0.001 | 0.014 | 0.021 | 0.025 | 0.028 | 0.030 | 0.042 | 0.059 | 0.75 | 6.34 |
| Ba T | <0.05 | DS | 0.44 | 68 | 0 | 0.026 | 0.008 | 32.73 | 0.006 | 0.009 | 0.019 | 0.026 | 0.030 | 0.037 | 0.038 | 0.042 | 0.049 | 0.35 | 2.75 |
| Ba T | <0.05 | RS | <0.05 | 68 | 0 | 0.040 | 0.041 | 100.51 | 0.006 | 0.011 | 0.024 | 0.0285 | 0.0355 | 0.065 | 0.091 | 0.154 | 0.294 | 4.40 | 26.60 |
| Ca D | 0.76 | DS | <0.05 | 68 | 0 | 2.76 | 1.50 | 54.19 | 0.81 | 0.22 | 1.7325 | 2.565 | 3.093 | 5.234 | 5.560 | 5.960 | 7.14 | 0.86 | 3.34 |
| Ca D | 0.76 | RS | <0.05 | 68 | 1.47 | 2.64 | 1.65 | 62.44 | 0.915 | <0.15 | 1.565 | 2.51 | 3.355 | 5.205 | 5.645 | 5.847 | 6.11 | 0.39 | 2.33 |
| Ca T | 0.14 | DS | <0.05 | 68 | 0 | 2.92 | 1.48 | 50.63 | 0.81 | 0.64 | 1.885 | 2.68 | 3.363 | 5.413 | 5.678 | 6.046 | 7.18 | 0.88 | 3.17 |
| Ca T | 0.14 | RS | <0.05 | 68 | 0 | 3.34 | 1.88 | 56.37 | 1.175 | 0.47 | 1.97 | 3.13 | 4.348 | 5.913 | 6.122 | 6.847 | 9.85 | 0.82 | 3.79 |
| TOC | <0.05 | DS | <0.05 | 68 | 1.47 | 1.58 | 0.59 | 37.46 | 0.3 | <0.5 | 1.2 | 1.5 | 1.8 | 2.23 | 2.73 | 3.032 | 4.3 | 1.87 | 9.45 |
| TOC | <0.05 | RS | <0.05 | 68 | 0 | 4.30 | 15.25 | 354.99 | 0.55 | 0.7 | 1.7 | 2.3 | 2.725 | 3.8 | 4.865 | 7.462 | 127.8 | 8.17 | 70.11 |
| Cl | 0.48 | DS | <0.05 | 68 | 52.94 | 1.67 | 1.99 | 119.14 | 0 | <1 | <1 | <1 | 2.7625 | 3.438 | 4.3965 | 5.409 | 13.5 | 3.48 | 20.84 |
| Cl | 0.48 | RS | <0.05 | 68 | 45.59 | 1.09 | 0.68 | 62.50 | 0.515 | <1 | <1 | 1.015 | 1.483 | 2.024 | 2.673 | 2.786 | 2.85 | 1.04 | 3.34 |
| Cu T | <0.05 | DS | <0.05 | 68 | 67.65 | 0.00 | 0.00 | 88.59 | 0 | <0.001 | <0.001 | <0.001 | 0.001 | 0.002 | 0.002 | 0.003 | 0.005 | 2.62 | 11.63 |
| Cu T | <0.05 | RS | <0.05 | 68 | 42.65 | 0.00 | 0.01 | 206.39 | 0.0005 | <0.001 | <0.001 | 0.001 | 0.003 | 0.006 | 0.0096 | 0.0243 | 0.043 | 4.65 | 27.31 |
| EC | 0.09 | DS | <0.05 | 68 | 0 | 68.04 | 124.83 | 183.47 | 11.6 | 8 | 38 | 48.9 | 63 | 81.8 | 104.65 | 165.82 | 1059 | 7.70 | 64.70 |
| EC | 0.09 | RS | <0.05 | 68 | 0 | 46.49 | 28.28 | 60.83 | 11.15 | 5 | 33.5 | 43.15 | 57.75 | 69 | 75.3 | 87.92 | 219 | 3.37 | 23.07 |
| Cr D | 0.05 | DS | <0.05 | 68 | 38.24 | 0.001 | 0.001 | 66.04 | 0.0005 | <0.001 | <0.001 | 0.001 | 0.002 | 0.0023 | 0.003 | 0.003 | 0.003 | 0.83 | 2.46 |
| Cr D | 0.05 | RS | <0.05 | 68 | 47.06 | 0.001 | 0.001 | 102.59 | 0.0005 | <0.001 | <0.001 | 0.001 | 0.001 | 0.002 | 0.002 | 0.004 | 0.008 | 4.64 | 30.13 |
| Cr T | <0.05 | DS | <0.05 | 68 | 26.47 | 0.002 | 0.001 | 66.62 | 0.0005 | <0.001 | <0.001 | 0.001 | 0.002 | 0.003 | 0.003 | 0.004 | 0.005 | 1.01 | 3.72 |
| Cr T | <0.05 | RS | <0.05 | 68 | 16.18 | 0.007 | 0.016 | 216.90 | 0.0015 | <0.001 | 0.001 | 0.002 | 0.007 | 0.015 | 0.0281 | 0.0426 | 0.119 | 5.50 | 38.75 |
| Sr D | 0.08 | DS | <0.05 | 68 | 0 | 0.029 | 0.016 | 57.39 | 0.0085 | 0.002 | 0.018 | 0.023 | 0.0365 | 0.0543 | 0.057 | 0.066 | 0.074 | 0.77 | 3.06 |
| Sr D | 0.08 | RS | <0.05 | 68 | 0 | 0.023 | 0.013 | 56.95 | 0.0095 | 0.001 | 0.015 | 0.021 | 0.031 | 0.042 | 0.043 | 0.046 | 0.05 | 0.21 | 2.21 |
| Sr T | 0.22 | DS | <0.05 | 68 | 0 | 0.041 | 0.079 | 193.77 | 0.0095 | 0.004 | 0.019 | 0.0265 | 0.047 | 0.0573 | 0.0643 | 0.074 | 0.671 | 7.67 | 64.52 |
| Sr T | 0.22 | RS | <0.05 | 68 | 0 | 0.029 | 0.019 | 65.85 | 0.0075 | 0.005 | 0.019 | 0.025 | 0.037 | 0.0473 | 0.05065 | 0.0543 | 0.144 | 3.50 | 22.85 |
| Fe D | <0.05 | DS | <0.05 | 68 | 2.94 | 0.34 | 0.14 | 42.48 | 0.08 | <0.1 | 0.2375 | 0.32 | 0.4 | 0.539 | 0.633 | 0.6764 | 0.69 | 0.59 | 3.20 |
| Fe D | <0.05 | RS | 0.05 | 68 | 8.82 | 0.72 | 0.39 | 54.03 | 0.25 | <0.1 | 0.3825 | 0.79 | 1.013 | 1.153 | 1.285 | 1.457 | 1.55 | -0.12 | 2.26 |
| Fe³⁺ | <0.05 | DS | 0.31 | 68 | 0 | 0.99 | 0.32 | 32.19 | 0.18 | 0.22 | 0.7975 | 0.965 | 1.155 | 1.439 | 1.579 | 1.663 | 1.96 | 0.42 | 3.90 |
| Fe³⁺ | <0.05 | RS | <0.05 | 68 | 0 | 4.14 | 5.69 | 137.46 | 0.925 | 0.78 | 1.8975 | 2.56 | 4.68 | 6.492 | 9.638 | 13.728 | 45.2 | 5.91 | 44.30 |
| Fe²⁺ | <0.05 | DS | <0.05 | 68 | 26.47 | 0.10 | 0.08 | 78.22 | 0.04 | <0.05 | <0.05 | 0.09 | 0.13 | 0.16 | 0.196 | 0.27 | 0.54 | 2.87 | 17.05 |
| Fe²⁺ | <0.05 | RS | <0.05 | 68 | 8.82 | 0.32 | 0.22 | 70.93 | 0.1 | <0.05 | 0.1975 | 0.3 | 0.4 | 0.489 | 0.62 | 0.994 | 1.33 | 2.08 | 10.33 |
| Fe T | <0.05 | DS | 0.56 | 68 | 0 | 1.09 | 0.32 | 29.17 | 0.185 | 0.32 | 0.8875 | 1.06 | 1.265 | 1.509 | 1.6 | 1.749 | 2.06 | 0.35 | 3.83 |
| Fe T | <0.05 | RS | <0.05 | 68 | 0 | 4.46 | 5.72 | 128.22 | 1.01 | 0.87 | 2.155 | 3.005 | 4.893 | 6.622 | 10.034 | 14.590 | 45.4 | 5.81 | 43.17 |
| P D | <0.05 | DS | <0.05 | 68 | 75.00 | 0.016 | 0.012 | 74.79 | 0 | <0.02 | <0.02 | <0.02 | 0.0125 | 0.033 | 0.04 | 0.04 | 0.07 | 2.14 | 8.01 |
| P D | <0.05 | RS | <0.05 | 68 | 41.18 | 0.035 | 0.044 | 124.87 | 0.015 | <0.02 | <0.02 | 0.025 | 0.04 | 0.063 | 0.093 | 0.1366 | 0.31 | 4.28 | 27.04 |
| P T | <0.05 | DS | <0.05 | 68 | 52.94 | 0.025 | 0.023 | 94.43 | 0 | <0.02 | <0.02 | <0.02 | 0.03 | 0.053 | 0.06 | 0.0732 | 0.15 | 2.75 | 14.18 |
| P T | <0.05 | RS | <0.05 | 68 | 2.94 | 0.11 | 0.13 | 117.51 | 0.035 | <0.02 | 0.04 | 0.065 | 0.115 | 0.233 | 0.2665 | 0.4826 | 0.82 | 3.51 | 18.75 |
| Li T | <0.05 | DS | <0.05 | 68 | 64.71 | 0.001 | 0.001 | 73.34 | 0 | <0.001 | <0.001 | <0.001 | 0.001 | 0.002 | 0.002 | 0.003 | 0.003 | 1.73 | 5.14 |
| Li T | <0.05 | RS | <0.05 | 68 | 50.00 | 0.001 | 0.001 | 100.30 | 0.00025 | <0.001 | <0.001 | 0.0008 | 0.002 | 0.003 | 0.004 | 0.006 | 0.007 | 2.56 | 10.95 |
| Mg D | 0.19 | DS | 0.16 | 68 | 0 | 1.23 | 0.57 | 46.64 | 0.39 | 0.15 | 0.825 | 1.08 | 1.668 | 1.969 | 2.200 | 2.370 | 2.68 | 0.35 | 2.50 |
| Mg D | 0.19 | RS | <0.05 | 68 | 1.47 | 1.07 | 0.59 | 55.47 | 0.58 | <0.1 | 0.5375 | 1.025 | 1.655 | 1.756 | 1.83 | 1.969 | 2.42 | -0.01 | 1.97 |
| Mg T | 0.73 | DS | <0.05 | 68 | 0 | 1.31 | 0.57 | 43.75 | 0.36 | 0.38 | 0.8575 | 1.16 | 1.765 | 2.050 | 2.312 | 2.423 | 2.83 | 0.45 | 2.47 |
| Mg T | 0.73 | RS | <0.05 | 68 | 0 | 1.32 | 0.59 | 44.40 | 0.41 | 0.29 | 0.905 | 1.25 | 1.8 | 2.079 | 2.137 | 2.424 | 2.62 | 0.12 | 2.15 |
| Mn D | 0.52 | DS | <0.05 | 68 | 33.82 | 0.032 | 0.032 | 98.78 | 0.01 | <0.02 | <0.02 | 0.02 | 0.04 | 0.07 | 0.07 | 0.08 | 0.23 | 3.78 | 24.19 |
| Mn D | 0.52 | RS | <0.05 | 68 | 38.24 | 0.044 | 0.061 | 136.35 | 0.02 | <0.02 | <0.02 | 0.03 | 0.0525 | 0.093 | 0.123 | 0.183 | 0.43 | 4.34 | 27.74 |
| Mn T | <0.05 | DS | <0.05 | 68 | 8.82 | 0.058 | 0.046 | 79.11 | 0.015 | <0.02 | 0.03 | 0.04 | 0.07 | 0.123 | 0.153 | 0.1732 | 0.25 | 1.85 | 7.00 |
| Mn T | <0.05 | RS | <0.05 | 68 | 0 | 0.23 | 0.52 | 228.24 | 0.04 | 0.03 | 0.0675 | 0.09 | 0.165 | 0.393 | 0.59 | 1.519 | 3.9 | 5.90 | 42.14 |
| Ni D | <0.05 | DS | 0.14 | 68 | 2.94 | 0.009 | 0.004 | 45.38 | 0.003 | <0.002 | 0.006 | 0.008 | 0.011 | 0.014 | 0.017 | 0.018 | 0.019 | 0.43 | 2.89 |
| Ni D | <0.05 | RS | <0.05 | 68 | 4.41 | 0.017 | 0.009 | 53.28 | 0.0075 | <0.002 | 0.009 | 0.019 | 0.024 | 0.029 | 0.031 | 0.031 | 0.037 | -0.17 | 2.07 |
| Ni T | <0.05 | DS | <0.05 | 68 | 0 | 0.028 | 0.010 | 33.90 | 0.007 | 0.01 | 0.021 | 0.027 | 0.034 | 0.038 | 0.044 | 0.048 | 0.063 | 0.80 | 4.63 |
| Ni T | <0.05 | RS | <0.05 | 68 | 0 | 0.11 | 0.17 | 146.90 | 0.0205 | 0.019 | 0.054 | 0.072 | 0.101 | 0.1751 | 0.2518 | 0.419 | 1.32 | 6.18 | 46.84 |
| NO₃ | 0.21 | DS | <0.05 | 68 | 1.47 | 0.35 | 0.35 | 100.85 | 0.08 | <0.02 | 0.1375 | 0.205 | 0.385 | 1.042 | 1.1665 | 1.230 | 1.32 | 1.59 | 4.24 |
| NO₃ | 0.21 | RS | <0.05 | 68 | 4.41 | 0.21 | 0.11 | 55.24 | 0.05 | <0.02 | 0.14 | 0.19 | 0.2525 | 0.343 | 0.36 | 0.4658 | 0.71 | 1.45 | 7.78 |
| NO₂ | <0.05 | DS | <0.05 | 68 | 33.82 | 0.04 | 0.03 | 83.34 | 0.025 | <0.01 | <0.01 | 0.035 | 0.06 | 0.09 | 0.0965 | 0.1 | 0.12 | 0.70 | 2.64 |
| NO₂ | <0.05 | RS | <0.05 | 68 | 73.53 | 0.01 | 0.01 | 141.14 | 0 | <0.01 | <0.01 | <0.01 | 0.01 | 0.023 | 0.0365 | 0.0466 | 0.1 | 4.28 | 25.25 |
| N T | <0.05 | DS | <0.05 | 68 | 1.47 | 2.96 | 3.21 | 108.40 | 0.6 | <0.3 | 1.275 | 1.65 | 3.2 | 5.84 | 10.56 | 14.492 | 15.2 | 2.58 | 9.63 |
| N T | <0.05 | RS | <0.05 | 68 | 16.18 | 1.04 | 1.38 | 132.82 | 0.2 | <0.3 | 0.3 | 0.5 | 0.95 | 2.9 | 3.395 | 5.628 | 7.2 | 2.69 | 10.80 |
| DO | <0.05 | DS | 0.22 | 68 | 0 | 7.68 | 1.43 | 18.66 | 1.065 | 4.82 | 6.68 | 7.48 | 8.61 | 9.889 | 9.973 | 10.596 | 12.1 | 0.55 | 3.27 |
| DO | <0.05 | RS | <0.05 | 68 | 0 | 5.61 | 1.44 | 25.76 | 1.185 | 3.22 | 4.4575 | 5.03 | 6.875 | 7.719 | 7.9 | 8.178 | 8.29 | 0.30 | 1.70 |
| pH | 0.06 | DS | <0.05 | 68 | 1.47 | 6.59 | 0.87 | 13.13 | 0.43 | <2 | 6.2975 | 6.64 | 7.11 | 7.303 | 7.493 | 7.576 | 7.67 | -4.07 | 28.67 |
| pH | 0.06 | RS | 0.28 | 68 | 0 | 6.84 | 0.37 | 5.34 | 0.29 | 6.05 | 6.5275 | 6.81 | 7.115 | 7.343 | 7.380 | 7.473 | 7.58 | 0.06 | 2.14 |
| K D | 0.34 | DS | <0.05 | 68 | 2.94 | 1.48 | 0.52 | 34.99 | 0.235 | <0.6 | 1.1475 | 1.525 | 1.703 | 1.858 | 1.9755 | 2.323 | 4.22 | 1.89 | 14.19 |
| K D | 0.34 | RS | <0.05 | 68 | 16.18 | 1.37 | 0.66 | 48.11 | 0.29 | <0.6 | 0.9175 | 1.45 | 1.703 | 2.098 | 2.299 | 2.650 | 3.41 | 0.13 | 3.44 |
| K T | 0.09 | DS | <0.05 | 68 | 1.47 | 1.58 | 0.58 | 36.50 | 0.245 | <0.6 | 1.29 | 1.6 | 1.783 | 1.98 | 2.069 | 2.409 | 5.06 | 3.15 | 22.61 |
| K T | 0.09 | RS | <0.05 | 68 | 1.47 | 1.69 | 0.52 | 30.73 | 0.265 | <0.6 | 1.4325 | 1.675 | 1.943 | 2.296 | 2.611 | 2.733 | 3.56 | 0.49 | 5.23 |
| Eh | <0.05 | DS | <0.05 | 68 | 0 | 92.09 | 63.26 | 68.70 | 23 | 11.6 | 64.15 | 84.55 | 110.25 | 134.3 | 155.7 | 173.62 | 515 | 4.54 | 32.79 |
| Eh | <0.05 | RS | <0.05 | 68 | 0 | 171.70 | 62.92 | 36.65 | 47 | 80.1 | 115.75 | 174.5 | 209 | 260.6 | 285.55 | 292.96 | 318 | 0.31 | 2.22 |
| SiO₂ | <0.05 | DS | <0.05 | 68 | 0 | 15.67 | 5.49 | 35.02 | 3 | 4.11 | 13.05 | 16.45 | 18.9 | 21.98 | 23.53 | 24.724 | 29 | -0.37 | 2.92 |
| SiO₂ | <0.05 | RS | 0.71 | 68 | 0 | 12.72 | 3.68 | 28.97 | 2.25 | 3.31 | 10.825 | 12.5 | 15.2 | 17.52 | 18.75 | 19.1 | 22.2 | -0.07 | 3.23 |
| Na D | <0.05 | DS | 0.05 | 68 | 0 | 3.86 | 1.64 | 42.54 | 0.985 | 0.54 | 2.755 | 3.665 | 4.83 | 6.068 | 6.592 | 8.047 | 8.55 | 0.68 | 3.64 |
| Na D | <0.05 | RS | <0.05 | 68 | 0 | 6.98 | 27.76 | 397.90 | 0.8 | 0.26 | 1.7725 | 2.675 | 3.463 | 4.376 | 4.719 | 59.957 | 216 | 6.94 | 53.51 |
| Na T | <0.05 | DS | <0.05 | 68 | 0 | 4.17 | 1.60 | 38.32 | 1.025 | 1.48 | 2.9775 | 3.9 | 5.07 | 6.353 | 6.794 | 8.524 | 9.34 | 1.03 | 4.52 |
| Na T | <0.05 | RS | <0.05 | 68 | 0 | 3.10 | 1.22 | 39.23 | 0.705 | 0.93 | 2.0825 | 3.025 | 3.678 | 5.000 | 5.369 | 5.519 | 5.97 | 0.46 | 2.58 |
| TDS | <0.05 | DS | 0.19 | 68 | 0 | 50.36 | 16.30 | 32.37 | 10.6 | 11.2 | 39.6 | 46.35 | 63 | 75.5 | 77.825 | 78.66 | 89 | 0.16 | 2.74 |
| TDS | <0.05 | RS | 0.67 | 68 | 0 | 57.35 | 16.14 | 28.15 | 12.15 | 22.4 | 45.225 | 57.4 | 69.375 | 79.36 | 82.455 | 84.912 | 96 | 0.05 | 2.40 |
| TSS | <0.05 | DS | <0.05 | 68 | 32.35 | 12.74 | 10.38 | 81.49 | 6.7 | <7 | <7 | 10.2 | 15.5 | 28 | 36.055 | 42.15 | 45 | 1.47 | 4.81 |
| TSS | <0.05 | RS | <0.05 | 68 | 0 | 112.36 | 202.96 | 180.64 | 24.5 | 8.5 | 21.8 | 41.95 | 95.5 | 213.3 | 347.95 | 971.66 | 1043 | 3.72 | 17.10 |
| ST | <0.05 | DS | 0.19 | 68 | 0 | 63.28 | 21.16 | 33.44 | 13.6 | 18.8 | 47.95 | 59.3 | 79 | 91.8 | 99.825 | 105.62 | 120 | 0.44 | 2.76 |
| ST | <0.05 | RS | <0.05 | 68 | 0 | 172.25 | 207.67 | 120.56 | 45 | 46 | 74.325 | 112 | 164.25 | 289.9 | 402.75 | 1043.76 | 1114 | 3.61 | 16.51 |
| SO₄²⁻ | 0.31 | DS | <0.05 | 68 | 73.53 | 1.90 | 3.03 | 159.19 | 0 | <1 | <1 | <1 | 1.715 | 6.648 | 7.951 | 9.4608 | 16.7 | 2.69 | 11.34 |
| SO₄²⁻ | 0.31 | RS | <0.05 | 68 | 77.94 | 0.94 | 0.94 | 99.92 | 0 | <1 | <1 | <1 | 0.5 | 2.454 | 2.966 | 3.752 | 4.35 | 2.09 | 6.55 |
| T | <0.05 | DS | <0.05 | 68 | 0 | 18.53 | 2.50 | 13.48 | 1.4 | 14.3 | 17.1 | 18.4 | 19.8 | 20.73 | 21.06 | 23.56 | 31.9 | 2.33 | 14.41 |
| T | <0.05 | RS | <0.05 | 68 | 0 | 24.39 | 2.74 | 11.22 | 1.5 | 20.3 | 22.5 | 24 | 25.625 | 27.23 | 27.865 | 30.048 | 38.5 | 2.26 | 12.57 |
| Ti D | <0.05 | DS | <0.05 | 68 | 73.53 | 0.0007 | 0.0005 | 63.87 | 0 | <0.001 | <0.001 | <0.001 | 0.001 | 0.001 | 0.002 | 0.002 | 0.003 | 3.10 | 14.21 |
| Ti D | <0.05 | RS | <0.05 | 68 | 55.88 | 0.002 | 0.003 | 202.68 | 0 | <0.001 | <0.001 | <0.001 | 0.002 | 0.003 | 0.003 | 0.005 | 0.025 | 6.91 | 55.81 |
| Ti T | <0.05 | DS | <0.05 | 68 | 39.71 | 0.003 | 0.005 | 179.04 | 0.0005 | <0.001 | <0.001 | 0.001 | 0.002 | 0.005 | 0.0112 | 0.019 | 0.025 | 3.54 | 15.96 |
| Ti T | <0.05 | RS | <0.05 | 68 | 14.71 | 0.040 | 0.092 | 230.63 | 0.00975 | <0.001 | 0.001 | 0.0105 | 0.042 | 0.085 | 0.153 | 0.294 | 0.638 | 4.95 | 31.73 |
| Turbidity | <0.05 | DS | <0.05 | 68 | 0 | 24.65 | 10.13 | 41.11 | 5.45 | 10.3 | 17.225 | 21.75 | 30.375 | 35.96 | 40.07 | 51.586 | 65.3 | 1.48 | 6.46 |
| Turbidity | <0.05 | RS | <0.05 | 68 | 0 | 155.54 | 287.48 | 184.82 | 33.05 | 14.3 | 47.075 | 70 | 121 | 297.7 | 438.45 | 1129.62 | 1890 | 4.50 | 25.56 |
| V T | <0.05 | DS | <0.05 | 68 | 77.94 | 0.0008 | 0.0007 | 85.27 | 0 | <0.001 | <0.001 | <0.001 | 0.0005 | 0.002 | 0.002 | 0.003 | 0.004 | 2.82 | 11.63 |
| V T | <0.05 | RS | <0.05 | 68 | 13.24 | 0.0069 | 0.0139 | 202.81 | 0.002 | <0.001 | 0.001 | 0.003 | 0.007 | 0.013 | 0.025 | 0.046 | 0.097 | 4.83 | 30.52 |

## MB-MLU

| **Parameter** | **pMW** | **Dataset** | **pSW** | **n** | **% <LD** | **µ** | **s** | **% CV** | **MAD** | **Min** | **P25** | **P50** | **P75** | **P90** | **P95** | **P98** | **Max** | **skewness** | **kurtosis** |
| --- | --- | --- | --- | --- | --- | --- | --- | --- | --- | --- | --- | --- | --- | --- | --- | --- | --- | --- | --- |
| Alk HCO₃ | <0.05 | DS | <0.05 | 92 | 0 | 28.89 | 16.78 | 58.09 | 7.6 | 5.48 | 18.525 | 23.7 | 35.75 | 55.57 | 59.88 | 80.406 | 92.6 | 1.72 | 6.05 |
| Alk HCO₃ | <0.05 | RS | <0.05 | 92 | 0 | 24.29 | 13.57 | 55.85 | 7.35 | 5.59 | 15.225 | 21.2 | 31.125 | 37.55 | 41.215 | 52.502 | 97.3 | 2.44 | 12.92 |
| Alk T | <0.05 | DS | <0.05 | 92 | 0 | 28.89 | 16.78 | 58.09 | 7.6 | 5.48 | 18.525 | 23.7 | 35.75 | 55.57 | 59.88 | 80.406 | 92.6 | 1.72 | 6.05 |
| Alk T | <0.05 | RS | <0.05 | 92 | 0 | 24.29 | 13.57 | 55.85 | 7.35 | 5.59 | 15.225 | 21.2 | 31.125 | 37.55 | 41.215 | 52.502 | 97.3 | 2.44 | 12.92 |
| Al D | <0.05 | DS | <0.05 | 92 | 72.83 | 0.02 | 0.02 | 96.66 | 0 | <0.02 | <0.02 | <0.02 | 0.02 | 0.03 | 0.04 | 0.0754 | 0.1 | 3.65 | 18.16 |
| Al D | <0.05 | RS | <0.05 | 92 | 44.57 | 0.04 | 0.05 | 122.87 | 0.01 | <0.02 | <0.02 | 0.02 | 0.0475 | 0.082 | 0.1265 | 0.201 | 0.25 | 2.75 | 11.61 |
| Al T | <0.05 | DS | <0.05 | 92 | 14.13 | 0.11 | 0.14 | 130.36 | 0.045 | <0.02 | 0.03 | 0.055 | 0.1225 | 0.217 | 0.3735 | 0.6116 | 0.72 | 2.89 | 12.16 |
| Al T | <0.05 | RS | <0.05 | 92 | 3.26 | 1.76 | 4.25 | 241.63 | 0.19 | <0.02 | 0.08 | 0.22 | 1.028 | 4.087 | 10.434 | 19.154 | 23.6 | 3.62 | 16.48 |
| Ba D | 0.08 | DS | <0.05 | 92 | 2.17 | 0.023 | 0.019 | 82.22 | 0.0075 | <0.001 | 0.011 | 0.017 | 0.027 | 0.0477 | 0.05635 | 0.069 | 0.123 | 2.35 | 11.55 |
| Ba D | 0.08 | RS | <0.05 | 92 | 0 | 0.024 | 0.013 | 53.55 | 0.005 | 0.003 | 0.017 | 0.022 | 0.027 | 0.039 | 0.0481 | 0.060 | 0.071 | 1.50 | 6.46 |
| Ba T | 0.27 | DS | <0.05 | 92 | 0 | 0.033 | 0.022 | 65.47 | 0.006 | 0.005 | 0.022 | 0.027 | 0.035 | 0.0596 | 0.0658 | 0.111 | 0.123 | 2.39 | 9.52 |
| Ba T | 0.27 | RS | <0.05 | 92 | 0 | 0.034 | 0.018 | 52.64 | 0.007 | 0.009 | 0.023 | 0.0285 | 0.040 | 0.0564 | 0.079 | 0.087 | 0.088 | 1.50 | 5.10 |
| Ca D | 0.05 | DS | <0.05 | 92 | 2.17 | 4.50 | 5.13 | 114.03 | 1.825 | <0.15 | 1.4025 | 3.175 | 5.463 | 10.47 | 12.105 | 13.736 | 38.7 | 3.64 | 23.89 |
| Ca D | 0.05 | RS | <0.05 | 92 | 0 | 4.93 | 4.49 | 91.03 | 1.585 | 0.2 | 2.465 | 3.75 | 6.648 | 9.527 | 9.886 | 12.326 | 36.8 | 4.27 | 30.95 |
| Ca T | 0.57 | DS | <0.05 | 92 | 0 | 5.85 | 5.95 | 101.69 | 1.6 | 0.33 | 2.8525 | 3.79 | 7.03 | 12.48 | 13.89 | 14.19 | 49.1 | 4.53 | 33.24 |
| Ca T | 0.57 | RS | <0.05 | 92 | 0 | 5.60 | 4.65 | 83.01 | 1.375 | 0.99 | 3.0775 | 3.96 | 7.11 | 10.13 | 10.855 | 16.374 | 36.9 | 3.86 | 25.64 |
| TOC | <0.05 | DS | <0.05 | 92 | 3.26 | 1.65 | 1.20 | 72.96 | 0.4 | <0.5 | 1 | 1.3 | 1.8 | 2.8 | 4.59 | 5.472 | 7.5 | 2.60 | 10.97 |
| TOC | <0.05 | RS | <0.05 | 92 | 1.09 | 3.11 | 2.49 | 79.91 | 0.9 | <0.5 | 1.425 | 2.2 | 4 | 6.75 | 9.3 | 9.642 | 10.6 | 1.60 | 4.75 |
| Cl | 0.95 | DS | <0.05 | 92 | 53.26 | 3.36 | 9.44 | 281.04 | 0 | <1 | <1 | <1 | 1.755 | 7.66 | 15.6 | 15.754 | 83.6 | 7.07 | 61.52 |
| Cl | 0.95 | RS | <0.05 | 92 | 51.09 | 2.20 | 5.65 | 256.62 | 0 | <1 | <1 | <1 | 1.678 | 4.648 | 5.976 | 11.624 | 50.8 | 7.48 | 65.98 |
| Cu T | <0.05 | DS | <0.05 | 92 | 80.43 | 0.0009 | 0.0019 | 206.73 | 0 | <0.001 | <0.001 | <0.001 | 0.0005 | 0.001 | 0.002 | 0.004 | 0.016 | 6.74 | 51.47 |
| Cu T | <0.05 | RS | <0.05 | 92 | 40.22 | 0.0021 | 0.0025 | 118.73 | 0.0005 | <0.001 | <0.001 | 0.001 | 0.003 | 0.005 | 0.0071 | 0.010 | 0.013 | 2.49 | 10.01 |
| EC | <0.05 | DS | <0.05 | 92 | 0 | 111.76 | 166.14 | 148.65 | 21.15 | 14.4 | 49.15 | 62 | 92.25 | 266.1 | 309.95 | 557.88 | 1246 | 4.83 | 30.69 |
| EC | <0.05 | RS | <0.05 | 92 | 0 | 70.43 | 90.52 | 128.53 | 21 | 10 | 32 | 46.5 | 78 | 113.1 | 124.55 | 250.88 | 802 | 6.32 | 51.86 |
| Cr D | <0.05 | DS | <0.05 | 92 | 59.78 | 0.001 | 0.001 | 84.63 | 0 | <0.001 | <0.001 | <0.001 | 0.001 | 0.002 | 0.003 | 0.003 | 0.004 | 2.40 | 8.43 |
| Cr D | <0.05 | RS | <0.05 | 92 | 84.78 | 0.001 | 0.0002 | 38.73 | 0 | <0.001 | <0.001 | <0.001 | 0.0005 | 0.001 | 0.001 | 0.001 | 0.002 | 3.76 | 21.21 |
| Cr T | 0.41 | DS | <0.05 | 92 | 46.74 | 0.001 | 0.001 | 90.91 | 0.0005 | <0.001 | <0.001 | 0.001 | 0.002 | 0.003 | 0.003 | 0.004 | 0.008 | 2.70 | 14.44 |
| Cr T | 0.41 | RS | <0.05 | 92 | 50.00 | 0.003 | 0.005 | 175.45 | 0 | <0.001 | <0.001 | <0.001 | 0.003 | 0.008 | 0.0121 | 0.023 | 0.026 | 3.15 | 13.18 |
| Sr D | 0.45 | DS | <0.05 | 92 | 1.09 | 0.038 | 0.046 | 121.23 | 0.0155 | <0.001 | 0.016 | 0.032 | 0.0455 | 0.0722 | 0.0939 | 0.1009 | 0.409 | 5.94 | 49.22 |
| Sr D | 0.45 | RS | <0.05 | 92 | 4.35 | 0.039 | 0.043 | 110.49 | 0.0095 | <0.001 | 0.022 | 0.032 | 0.041 | 0.074 | 0.0791 | 0.095 | 0.393 | 6.25 | 52.78 |
| Sr T | <0.05 | DS | <0.05 | 92 | 0 | 0.073 | 0.165 | 227.90 | 0.0105 | 0.008 | 0.034 | 0.04 | 0.0545 | 0.0973 | 0.1046 | 0.4668 | 1.46 | 7.18 | 59.85 |
| Sr T | <0.05 | RS | <0.05 | 92 | 0 | 0.043 | 0.044 | 101.56 | 0.008 | 0.012 | 0.026 | 0.0335 | 0.042 | 0.076 | 0.086 | 0.098 | 0.407 | 6.53 | 55.69 |
| Fe D | <0.05 | DS | <0.05 | 92 | 22.83 | 0.24 | 0.23 | 95.45 | 0.11 | <0.1 | 0.11 | 0.17 | 0.3 | 0.389 | 0.6385 | 1.0544 | 1.17 | 2.57 | 10.47 |
| Fe D | <0.05 | RS | <0.05 | 92 | 9.78 | 0.54 | 0.39 | 72.65 | 0.215 | <0.1 | 0.295 | 0.45 | 0.7175 | 0.862 | 1.251 | 1.875 | 2.15 | 1.68 | 7.37 |
| Fe³⁺ | <0.05 | DS | <0.05 | 92 | 2.17 | 1.02 | 0.51 | 49.81 | 0.215 | <0.05 | 0.775 | 1 | 1.143 | 1.711 | 1.928 | 2.143 | 3.48 | 1.40 | 8.39 |
| Fe³⁺ | <0.05 | RS | <0.05 | 92 | 2.17 | 3.06 | 3.52 | 115.14 | 1.05 | <0.05 | 1.29 | 2.065 | 3.533 | 6.294 | 9.729 | 17.254 | 19.1 | 2.93 | 12.65 |
| Fe²⁺ | <0.05 | DS | <0.05 | 92 | 35.87 | 0.11 | 0.14 | 126.96 | 0.055 | <0.05 | <0.05 | 0.08 | 0.13 | 0.209 | 0.317 | 0.419 | 1.06 | 4.31 | 27.77 |
| Fe²⁺ | <0.05 | RS | <0.05 | 92 | 13.04 | 0.27 | 0.33 | 122.29 | 0.1 | <0.05 | 0.1 | 0.19 | 0.3 | 0.46 | 0.7355 | 1.1004 | 2.58 | 4.46 | 30.06 |
| Fe T | <0.05 | DS | <0.05 | 92 | 2.17 | 1.13 | 0.59 | 52.27 | 0.2 | <0.1 | 0.8375 | 1.09 | 1.223 | 1.808 | 2.285 | 2.660 | 3.56 | 1.38 | 6.96 |
| Fe T | <0.05 | RS | <0.05 | 92 | 2.17 | 3.33 | 3.64 | 109.47 | 1.155 | <0.1 | 1.5 | 2.365 | 3.753 | 6.724 | 10.206 | 18.166 | 19.9 | 2.90 | 12.58 |
| P D | <0.05 | DS | <0.05 | 92 | 78.26 | 0.031 | 0.063 | 206.23 | 0 | <0.02 | <0.02 | <0.02 | 0.01 | 0.086 | 0.1445 | 0.212 | 0.47 | 4.69 | 30.11 |
| P D | <0.05 | RS | <0.05 | 92 | 46.74 | 0.033 | 0.072 | 214.50 | 0.01 | <0.02 | <0.02 | 0.02 | 0.03 | 0.05 | 0.07 | 0.184 | 0.5 | 5.99 | 40.03 |
| P T | <0.05 | DS | <0.05 | 92 | 66.30 | 0.058 | 0.125 | 216.78 | 0 | <0.02 | <0.02 | <0.02 | 0.03 | 0.201 | 0.3225 | 0.5636 | 0.64 | 3.30 | 13.79 |
| P T | <0.05 | RS | <0.05 | 92 | 15.22 | 0.097 | 0.235 | 243.57 | 0.025 | <0.02 | 0.03 | 0.04 | 0.0875 | 0.141 | 0.2265 | 0.4642 | 2.04 | 7.02 | 57.97 |
| Li T | 0.57 | DS | <0.05 | 92 | 55.43 | 0.001 | 0.001 | 105.28 | 0 | <0.001 | <0.001 | <0.001 | 0.002 | 0.002 | 0.002 | 0.003 | 0.01 | 5.18 | 40.60 |
| Li T | 0.57 | RS | <0.05 | 92 | 56.52 | 0.001 | 0.001 | 99.86 | 0 | <0.001 | <0.001 | <0.001 | 0.001 | 0.002 | 0.003 | 0.004 | 0.006 | 3.02 | 13.64 |
| Mg D | 0.53 | DS | <0.05 | 92 | 3.26 | 1.69 | 1.24 | 73.59 | 0.855 | <0.1 | 0.61 | 1.405 | 2.4 | 3.436 | 3.915 | 4.423 | 4.74 | 0.64 | 2.42 |
| Mg D | 0.53 | RS | <0.05 | 92 | 2.17 | 1.69 | 0.97 | 57.42 | 0.685 | <0.1 | 0.995 | 1.55 | 2.48 | 3.007 | 3.294 | 3.501 | 3.8 | 0.23 | 2.18 |
| Mg T | 0.38 | DS | <0.05 | 92 | 0 | 2.19 | 1.25 | 57.21 | 0.76 | 0.3 | 1.285 | 1.855 | 3.05 | 3.767 | 4.497 | 5.455 | 6.02 | 1.04 | 3.79 |
| Mg T | 0.38 | RS | <0.05 | 92 | 0 | 1.95 | 0.92 | 46.97 | 0.63 | 0.43 | 1.21 | 1.7 | 2.66 | 3.233 | 3.586 | 3.857 | 3.91 | 0.52 | 2.15 |
| Mn D | <0.05 | DS | <0.05 | 92 | 16.30 | 0.14 | 0.32 | 221.90 | 0.04 | <0.02 | 0.0275 | 0.06 | 0.1025 | 0.277 | 0.613 | 0.969 | 2.54 | 5.44 | 38.97 |
| Mn D | <0.05 | RS | <0.05 | 92 | 9.78 | 0.16 | 0.25 | 163.42 | 0.045 | <0.02 | 0.04 | 0.075 | 0.18 | 0.303 | 0.4685 | 0.722 | 1.97 | 5.04 | 34.53 |
| Mn T | <0.05 | DS | <0.05 | 92 | 1.09 | 0.24 | 0.50 | 207.49 | 0.045 | <0.02 | 0.06 | 0.095 | 0.18 | 0.454 | 0.963 | 1.431 | 3.51 | 4.94 | 30.65 |
| Mn T | <0.05 | RS | <0.05 | 92 | 1.09 | 0.30 | 0.41 | 137.34 | 0.085 | <0.02 | 0.1 | 0.175 | 0.3275 | 0.496 | 1.138 | 1.641 | 2.67 | 3.78 | 19.50 |
| Ni D | <0.05 | DS | <0.05 | 92 | 23.91 | 0.006 | 0.006 | 104.15 | 0.003 | <0.002 | 0.002 | 0.004 | 0.007 | 0.010 | 0.016 | 0.026 | 0.032 | 2.51 | 10.44 |
| Ni D | <0.05 | RS | <0.05 | 92 | 5.43 | 0.014 | 0.009 | 66.78 | 0.0045 | <0.002 | 0.008 | 0.0125 | 0.018 | 0.0221 | 0.028 | 0.041 | 0.049 | 1.51 | 7.08 |
| Ni T | <0.05 | DS | <0.05 | 92 | 3.26 | 0.025 | 0.013 | 51.76 | 0.006 | <0.002 | 0.018 | 0.025 | 0.030 | 0.037 | 0.043 | 0.057 | 0.074 | 0.93 | 6.05 |
| Ni T | <0.05 | RS | <0.05 | 92 | 0 | 0.078 | 0.079 | 102.00 | 0.0245 | 0.002 | 0.0365 | 0.057 | 0.082 | 0.1548 | 0.248 | 0.356 | 0.455 | 2.68 | 11.32 |
| NO₃ | 0.88 | DS | <0.05 | 92 | 4.35 | 0.49 | 1.87 | 382.90 | 0.08 | <0.02 | 0.07 | 0.14 | 0.26 | 0.56 | 1.783 | 2.373 | 17.6 | 8.65 | 82.40 |
| NO₃ | 0.88 | RS | <0.05 | 92 | 3.26 | 0.33 | 1.18 | 354.65 | 0.065 | <0.02 | 0.08 | 0.145 | 0.2275 | 0.487 | 0.9125 | 0.99816 | 11.2 | 8.98 | 86.40 |
| NO₂ | <0.05 | DS | <0.05 | 92 | 35.87 | 0.06 | 0.15 | 238.23 | 0.015 | <0.01 | <0.01 | 0.02 | 0.04 | 0.079 | 0.3345 | 0.5944 | 0.99 | 4.25 | 22.59 |
| NO₂ | <0.05 | RS | <0.05 | 92 | 64.13 | 0.021 | 0.038 | 178.18 | 0 | <0.01 | <0.01 | <0.01 | 0.02 | 0.06 | 0.08 | 0.171 | 0.21 | 3.58 | 17.20 |
| N T | <0.05 | DS | <0.05 | 92 | 16.30 | 2.45 | 2.43 | 99.10 | 1.3 | <0.3 | 0.825 | 1.7 | 3.1 | 5.89 | 6.615 | 8.592 | 12.8 | 1.92 | 8.02 |
| N T | <0.05 | RS | <0.05 | 92 | 8.70 | 1.28 | 1.70 | 132.35 | 0.4 | <0.3 | 0.5 | 0.8 | 1.3 | 2.63 | 4.165 | 5.644 | 13.1 | 4.47 | 29.83 |
| DO | <0.05 | DS | <0.05 | 92 | 0 | 6.56 | 1.20 | 18.38 | 0.615 | 2.08 | 6.0575 | 6.585 | 7.233 | 7.728 | 8.204 | 8.561 | 9.82 | -1.01 | 6.28 |
| DO | <0.05 | RS | <0.05 | 92 | 1.09 | 4.53 | 0.90 | 19.92 | 0.565 | <2 | 3.8925 | 4.43 | 5.003 | 5.675 | 6.219 | 6.629 | 6.9 | -0.09 | 5.22 |
| pH | <0.05 | DS | <0.05 | 92 | 0 | 7.34 | 0.42 | 5.68 | 0.24 | 5.8 | 7.1175 | 7.3 | 7.585 | 7.848 | 7.989 | 8.3218 | 8.46 | -0.08 | 4.77 |
| pH | <0.05 | RS | <0.05 | 92 | 0 | 6.96 | 0.48 | 6.94 | 0.265 | 5.72 | 6.6375 | 6.95 | 7.15 | 7.523 | 8.008 | 8.195 | 8.45 | 0.76 | 4.40 |
| K D | <0.05 | DS | <0.05 | 92 | 48.91 | 1.15 | 2.75 | 238.27 | 0.31 | <0.6 | <0.6 | 0.61 | 0.92 | 1.806 | 4.595 | 5.477 | 24.8 | 7.33 | 64.51 |
| K D | <0.05 | RS | <0.05 | 92 | 27.17 | 1.14 | 1.88 | 164.71 | 0.51 | <0.6 | <0.6 | 0.835 | 1.248 | 2.181 | 2.262 | 2.559 | 17.8 | 7.97 | 73.70 |
| K T | 0.39 | DS | <0.05 | 92 | 14.13 | 1.65 | 3.34 | 202.77 | 0.265 | <0.6 | 0.6975 | 0.95 | 1.253 | 3.885 | 5.289 | 5.834 | 30.7 | 7.52 | 67.53 |
| K T | 0.39 | RS | <0.05 | 92 | 15.22 | 1.44 | 2.04 | 141.44 | 0.385 | <0.6 | 0.7 | 1.045 | 1.56 | 2.471 | 2.891 | 3.548 | 18.9 | 7.27 | 64.76 |
| Eh | <0.05 | DS | 0.30 | 92 | 0 | 114.42 | 44.12 | 38.56 | 23.75 | 17.3 | 89.275 | 114.5 | 135.75 | 162.8 | 186.05 | 214.26 | 251 | 0.28 | 3.71 |
| Eh | <0.05 | RS | <0.05 | 92 | 0 | 154.68 | 47.76 | 30.88 | 23 | -20.7 | 133 | 159 | 178 | 210.2 | 223.1 | 248.52 | 270 | -0.52 | 4.52 |
| SiO₂ | <0.05 | DS | <0.05 | 92 | 0 | 11.76 | 4.34 | 36.92 | 3.09 | 2.82 | 8.36 | 13.15 | 15.1 | 16.68 | 17.625 | 18.336 | 18.7 | -0.44 | 2.05 |
| SiO₂ | <0.05 | RS | 0.18 | 92 | 0 | 10.06 | 2.83 | 28.09 | 1.84 | 2.69 | 8.28 | 10.25 | 11.8 | 13.81 | 14.31 | 14.876 | 16.2 | -0.44 | 3.16 |
| Na D | 0.83 | DS | <0.05 | 92 | 0 | 4.94 | 15.11 | 305.55 | 0.745 | 0.25 | 1.2575 | 2.07 | 2.765 | 5.871 | 18.535 | 27.872 | 139 | 7.96 | 72.85 |
| Na D | 0.83 | RS | <0.05 | 92 | 0 | 5.15 | 16.78 | 325.63 | 0.455 | 0.2 | 1.6625 | 2.075 | 2.56 | 6.691 | 9.322 | 36.396 | 121 | 6.43 | 44.19 |
| Na T | <0.05 | DS | <0.05 | 92 | 0 | 6.58 | 17.06 | 259.34 | 0.61 | 0.37 | 2.46 | 2.91 | 3.718 | 15.81 | 24.24 | 28.636 | 158 | 7.94 | 72.79 |
| Na T | <0.05 | RS | <0.05 | 92 | 0 | 5.78 | 18.14 | 313.93 | 0.575 | 0.46 | 1.8425 | 2.385 | 2.985 | 7.469 | 10.565 | 36.992 | 137 | 6.52 | 45.87 |
| TDS | 0.99 | DS | <0.05 | 92 | 0 | 62.53 | 67.04 | 107.21 | 7.85 | 22.7 | 39.675 | 45.45 | 59.625 | 98 | 147 | 149.72 | 631 | 6.98 | 60.82 |
| TDS | 0.99 | RS | <0.05 | 92 | 0 | 58.52 | 55.15 | 94.24 | 13 | 16 | 38 | 46.75 | 64 | 78.1 | 89.2 | 141.34 | 525 | 7.08 | 61.78 |
| TSS | <0.05 | DS | <0.05 | 92 | 13.04 | 17.16 | 11.05 | 64.42 | 6.8 | <7 | 9 | 14.35 | 24.075 | 31 | 35.45 | 43.94 | 56 | 1.04 | 4.16 |
| TSS | <0.05 | RS | <0.05 | 92 | 7.61 | 54.83 | 85.91 | 156.70 | 17.25 | <7 | 13 | 27.6 | 49.5 | 127.4 | 241.4 | 326.12 | 523 | 3.27 | 15.06 |
| ST | <0.05 | DS | <0.05 | 92 | 0 | 79.91 | 70.53 | 88.26 | 12.5 | 32.3 | 51.6 | 63 | 77.375 | 139.3 | 175.25 | 184.54 | 654 | 6.25 | 51.77 |
| ST | <0.05 | RS | <0.05 | 92 | 0 | 115.02 | 100.32 | 87.22 | 28.9 | 32.4 | 58.25 | 77.6 | 127 | 227.8 | 326.15 | 440.18 | 576 | 2.66 | 11.00 |
| SO₄²⁻ | 0.78 | DS | <0.05 | 92 | 75.00 | 6.08 | 31.49 | 517.59 | 0 | <1 | <1 | <1 | 0.635 | 7.714 | 23.85 | 31.258 | 298 | 8.96 | 86.57 |
| SO₄²⁻ | 0.78 | RS | <0.05 | 92 | 70.65 | 4.62 | 23.50 | 508.36 | 0 | <1 | <1 | <1 | 1.25 | 8.721 | 11.255 | 19.018 | 221 | 8.98 | 86.25 |
| T | <0.05 | DS | 0.58 | 92 | 0 | 18.73 | 2.34 | 12.48 | 1.7 | 13.4 | 17.175 | 18.6 | 20.625 | 21.6 | 22.28 | 23.9 | 24.2 | 0.17 | 2.65 |
| T | <0.05 | RS | <0.05 | 92 | 0 | 25.34 | 2.23 | 8.82 | 1.45 | 21.2 | 23.625 | 25.25 | 26.5 | 28.67 | 29.71 | 30 | 31.8 | 0.52 | 3.07 |
| Ti D | 0.19 | DS | <0.05 | 92 | 77.17 | 0.001 | 0.001 | 70.79 | 0 | <0.001 | <0.001 | <0.001 | 0.0005 | 0.001 | 0.002 | 0.002 | 0.003 | 3.01 | 12.42 |
| Ti D | 0.19 | RS | <0.05 | 92 | 69.57 | 0.001 | 0.001 | 124.75 | 0 | <0.001 | <0.001 | <0.001 | 0.001 | 0.0021 | 0.003 | 0.005 | 0.009 | 3.59 | 18.87 |
| Ti T | <0.05 | DS | <0.05 | 92 | 41.30 | 0.003 | 0.004 | 162.56 | 0.0005 | <0.001 | <0.001 | 0.001 | 0.002 | 0.0069 | 0.009 | 0.016 | 0.028 | 3.76 | 19.72 |
| Ti T | <0.05 | RS | <0.05 | 92 | 22.83 | 0.020 | 0.045 | 219.88 | 0.0035 | <0.001 | 0.001 | 0.004 | 0.016 | 0.069 | 0.091 | 0.189 | 0.244 | 3.61 | 17.09 |
| Turbidity | <0.05 | DS | <0.05 | 92 | 0 | 23.34 | 9.68 | 41.48 | 5.3 | 4.84 | 16.375 | 21.6 | 29.375 | 35.5 | 40.94 | 45.726 | 56.4 | 0.74 | 3.86 |
| Turbidity | <0.05 | RS | <0.05 | 92 | 0 | 68.83 | 77.13 | 112.05 | 14.95 | 3.44 | 31.5 | 44.4 | 64.35 | 131.8 | 228.7 | 284.34 | 520 | 3.31 | 16.99 |
| V T | <0.05 | DS | <0.05 | 92 | 79.35 | 0.001 | 0.0005 | 70.29 | 0 | <0.001 | <0.001 | <0.001 | 0.0005 | 0.001 | 0.002 | 0.002 | 0.003 | 3.30 | 14.50 |
| V T | <0.05 | RS | <0.05 | 92 | 36.96 | 0.004 | 0.007 | 173.71 | 0.0005 | <0.001 | <0.001 | 0.001 | 0.004 | 0.010 | 0.015 | 0.031 | 0.038 | 3.41 | 15.86 |

# Appendix B – UTL methods

The tables compile the Upper Tolerance Limit (UTL) values calculated for different geochemical parameters in surface waters, organized by classes and considering the dry and rainy periods separately, as well as the assumed statistical distributions and their respective confidence levels. In general, all limits were estimated with 95% coverage and 95% confidence, indicating that the UTL values represent concentrations above which only a small fraction of the data is expected, and are therefore suitable as upper reference limits for anomaly assessment.

## MB-MLU

| **Parameter** | **Season** | **UTL** | **Distribution** | **Confidence level** |
| --- | --- | --- | --- | --- |
| Al T | Dry | 0.72 | Data do not follow a Discernible Distribution | 95% UTL with 95% Coverage |
| Al T | Rainy | 25.10 | Data do not follow a Discernible Distribution | 95% UTL with 95% Coverage |
| Ba D | Dry | 0.07 | Background Statistics Assuming Gamma Distribution | 95% WH Approx. Gamma UTL with 95% Coverage |
| Ba D | Rainy | 0.07 | Data do not follow a Discernible Distribution | 95% UTL with 95% Coverage |
| Ba T | Dry | 0.12 | Data do not follow a Discernible Distribution | 95% UTL with 95% Coverage |
| Ba T | Rainy | 0.15 | Data do not follow a Discernible Distribution | 95% UTL with 95% Coverage |
| Fe D | Dry | 1.17 | Data do not follow a Discernible Distribution | 95% UTL with 95% Coverage |
| Fe D | Rainy | 1.64 | Background Statistics Assuming Gamma Distribution | 95% WH Approx. Gamma UTL with 95% Coverage |
| Fe T | Dry | 3.56 | Data do not follow a Discernible Distribution | 95% UTL with 95% Coverage |
| Fe T | Rainy | 19.90 | Data do not follow a Discernible Distribution | 95% UTL with 95% Coverage |
| Mn D | Dry | 2.54 | Data do not follow a Discernible Distribution | 95% UTL with 95% Coverage |
| Mn D | Rainy | 1.19 | Data do not follow a Discernible Distribution | 95% UTL with 95% Coverage |
| Mn T | Dry | 3.51 | Data do not follow a Discernible Distribution | 95% UTL with 95% Coverage |
| Mn T | Rainy | 0.97 | Background Statistics assuming Lognormal Distribution | 95% UTL with 95% Coverage |
| Ni D | Dry | 0.03 | Data do not follow a Discernible Distribution | 95% UTL with 95% Coverage |
| Ni D | Rainy | 0.03 | Background Statistics Assuming Normal Distribution | 95% UTL with 95% Coverage |
| Ni T | Dry | 0.05 | Background Statistics Assuming Normal Distribution | 95% UTL with 95% Coverage |
| Ni T | Rainy | 0.46 | Data do not follow a Discernible Distribution | 95% UTL with 95% Coverage |
| Sr D | Dry | 0.41 | Data do not follow a Discernible Distribution | 95% UTL with 95% Coverage |
| Sr D | Rainy | 0.39 | Data do not follow a Discernible Distribution | 95% UTL with 95% Coverage |
| Sr T | Dry | 1.46 | Data do not follow a Discernible Distribution | 95% UTL with 95% Coverage |
| Sr T | Rainy | 0.10 | Background Statistics assuming Lognormal Distribution | 95% UTL with 95% Coverage |

TTG-P

| **Parameter** | **Season** | **UTL** | **Distribution** | **Confidence level** |
| --- | --- | --- | --- | --- |
| Al T | Dry | 0.59 | Background Statistics assuming Lognormal Distribution | 95% UTL with 95% Coverage |
| Ba D | Dry | 0.04 | Background Statistics Assuming Normal Distribution | 95% UTL with 95% Coverage |
| Ba T | Dry | 0.04 | Background Statistics Assuming Normal Distribution | 95% UTL with 95% Coverage |
| Fe D | Dry | 0.62 | Background Statistics Assuming Normal Distribution | 95% UTL with 95% Coverage |
| Fe T | Dry | 1.72 | Background Statistics Assuming Normal Distribution | 95% UTL with 95% Coverage |
| Mn D | Dry | 0.23 | Data do not follow a Discernible Distribution | 95% UTL with 95% Coverage |
| Mn T | Dry | 0.25 | Data do not follow a Discernible Distribution | 95% UTL with 95% Coverage |
| Ni D | Dry | 0.02 | Background Statistics Assuming Normal Distribution | 95% UTL with 95% Coverage |
| Ni T | Dry | 0.05 | Background Statistics Assuming Normal Distribution | 95% UTL with 95% Coverage |
| Sr D | Dry | 0.07 | Background Statistics Assuming Gamma Distribution | 95% WH Approx. Gamma UTL with 95% Coverage |
| Sr T | Dry | 0.67 | Data do not follow a Discernible Distribution | 95% UTL with 95% Coverage |
| Al T | Rainy | 20.23 | Background Statistics assuming Lognormal Distribution | 95% UTL with 95% Coverage |
| Ba D | Rainy | 0.07 | Data do not follow a Discernible Distribution | 95% UTL with 95% Coverage |
| Ba T | Rainy | 0.29 | Data do not follow a Discernible Distribution | 95% UTL with 95% Coverage |
| Fe D | Rainy | 1.45 | Background Statistics Assuming Normal Distribution | 95% UTL with 95% Coverage |
| Fe T | Rainy | 45.40 | Data do not follow a Discernible Distribution | 95% UTL with 95% Coverage |
| Mn D | Rainy | 1.97 | Data do not follow a Discernible Distribution | 95% UTL with 95% Coverage |
| Mn T | Rainy | 3.90 | Data do not follow a Discernible Distribution | 95% UTL with 95% Coverage |
| Ni D | Rainy | 0.03 | Background Statistics Assuming Normal Distribution | 95% UTL with 95% Coverage |
| Ni T | Rainy | 1.32 | Data do not follow a Discernible Distribution | 95% UTL with 95% Coverage |
| Sr D | Rainy | 0.06 | Background Statistics Assuming Normal Distribution | 95% UTL with 95% Coverage |
| Sr T | Rainy | 0.08 | Background Statistics Assuming Gamma Distribution | 95% WH Approx. Gamma UTL with 95% Coverage |

MVS-MLU

| **Parameter** | **Season** | **UTL** | **Distribution** | **Confidence level** |
| --- | --- | --- | --- | --- |
| Al T | Dry | 0.46 | Background Statistics Assuming Gamma Distribution | 95% WH Approx. Gamma UTL with 95% Coverage |
| Ba D | Dry | 0.06 | Background Statistics Assuming Gamma Distribution | 95% WH Approx. Gamma UTL with 95% Coverage |
| Ba T | Dry | 0.07 | Background Statistics Assuming Gamma Distribution | 95% WH Approx. Gamma UTL with 95% Coverage |
| Fe D | Dry | 0.85 | Background Statistics Assuming Normal Distribution | 95% UTL with 95% Coverage |
| Fe T | Dry | 4.05 | Data do not follow a Discernible Distribution | 95% UTL with 95% Coverage |
| Mn D | Dry | 0.68 | Background Statistics assuming Lognormal Distribution | 95% UTL with 95% Coverage |
| Mn T | Dry | 1.03 | Data do not follow a Discernible Distribution | 95% UTL with 95% Coverage |
| Ni D | Dry | 0.03 | Background Statistics Assuming Gamma Distribution | 95% WH Approx. Gamma UTL with 95% Coverage |
| Ni T | Dry | 0.06 | Background Statistics Assuming Gamma Distribution | 95% WH Approx. Gamma UTL with 95% Coverage |
| Sr D | Dry | 0.11 | Background Statistics Assuming Gamma Distribution | 95% WH Approx. Gamma UTL with 95% Coverage |
| Sr T | Dry | 0.12 | Background Statistics Assuming Gamma Distribution | 95% WH Approx. Gamma UTL with 95% Coverage |
| Al T | Rainy | 5.51 | Data do not follow a Discernible Distribution | 95% UTL with 95% Coverage |
| Ba D | Rainy | 0.07 | Data do not follow a Discernible Distribution | 95% UTL with 95% Coverage |
| Ba T | Rainy | 0.07 | Background Statistics Assuming Gamma Distribution | 95% WH Approx. Gamma UTL with 95% Coverage |
| Fe D | Rainy | 1.21 | Background Statistics Assuming Normal Distribution | 95% UTL with 95% Coverage |
| Fe T | Rainy | 5.93 | Background Statistics Assuming Gamma Distribution | 95% WH Approx. Gamma UTL with 95% Coverage |
| Mn D | Rainy | 0.60 | Background Statistics assuming Lognormal Distribution | 95% UTL with 95% Coverage |
| Mn T | Rainy | 0.65 | Background Statistics Assuming Gamma Distribution | 95% WH Approx. Gamma UTL with 95% Coverage |
| Ni D | Rainy | 0.03 | Background Statistics Assuming Normal Distribution | 95% UTL with 95% Coverage |
| Ni T | Rainy | 0.14 | Background Statistics Assuming Gamma Distribution | 95% WH Approx. Gamma UTL with 95% Coverage |
| Sr D | Rainy | 0.11 | Background Statistics assuming Lognormal Distribution | 95% UTL with 95% Coverage |
| Sr T | Rainy | 0.12 | Background Statistics assuming Lognormal Distribution | 95% UTL with 95% Coverage |

TTG-UP

| **Parameter** | **Season** | **UTL** | **Distribution** | **Confidence level** |
| --- | --- | --- | --- | --- |
| Al T | Dry | 1.48 | Background Statistics assuming Lognormal Distribution | 95% UTL with 95% Coverage |
| Ba D | Dry | 0.18 | Background Statistics assuming Lognormal Distribution | 95% UTL with 95% Coverage |
| Ba T | Dry | 0.88 | Data do not follow a Discernible Distribution | 95% UTL with 95% Coverage |
| Fe D | Dry | 1.37 | Background Statistics Assuming Gamma Distribution | 95% WH Approx. Gamma UTL with 95% Coverage |
| Fe T | Dry | 5.01 | Background Statistics assuming Lognormal Distribution | 95% UTL with 95% Coverage |
| Mn D | Dry | 0.35 | Background Statistics assuming Lognormal Distribution | 95% UTL with 95% Coverage |
| Mn T | Dry | 0.39 | Background Statistics Assuming Gamma Distribution | 95% WH Approx. Gamma UTL with 95% Coverage |
| Ni D | Dry | 0.05 | Data do not follow a Discernible Distribution | 95% UTL with 95% Coverage |
| Ni T | Dry | 0.12 | Background Statistics assuming Lognormal Distribution | 95% UTL with 95% Coverage |
| Sr D | Dry | 0.32 | Background Statistics assuming Lognormal Distribution | 95% UTL with 95% Coverage |
| Sr T | Dry | 1.17 | Data do not follow a Discernible Distribution | 95% UTL with 95% Coverage |
| Al T | Rainy | 13.43 | Background Statistics assuming Lognormal Distribution | 95% UTL with 95% Coverage |
| Ba D | Rainy | 0.10 | Background Statistics Assuming Normal Distribution | 95% UTL with 95% Coverage |
| Ba T | Rainy | 0.12 | Background Statistics Assuming Normal Distribution | 95% UTL with 95% Coverage |
| Fe D | Rainy | 1.82 | Background Statistics Assuming Normal Distribution | 95% UTL with 95% Coverage |
| Fe T | Rainy | 11.09 | Background Statistics assuming Lognormal Distribution | 95% UTL with 95% Coverage |
| Mn D | Rainy | 0.51 | Data do not follow a Discernible Distribution | 95% UTL with 95% Coverage |
| Mn T | Rainy | 1.45 | Data do not follow a Discernible Distribution | 95% UTL with 95% Coverage |
| Ni D | Rainy | 0.04 | Background Statistics Assuming Normal Distribution | 95% UTL with 95% Coverage |
| Ni T | Rainy | 0.24 | Background Statistics assuming Lognormal Distribution | 95% UTL with 95% Coverage |
| Sr D | Rainy | 0.22 | Background Statistics Assuming Gamma Distribution | 95% WH Approx. Gamma UTL with 95% Coverage |
| Sr T | Rainy | 0.23 | Background Statistics Assuming Gamma Distribution | 95% WH Approx. Gamma UTL with 95% Coverage |

# Appendix C – Spatial distribution maps

Spatial distribution maps of the main metals during the rainy and dry seasons in the study area, along with box plots for each class.
